# Pharmacological inhibition of tumor anabolism and host catabolism as a cancer therapy

**DOI:** 10.1038/s41598-021-84538-6

**Published:** 2021-03-04

**Authors:** Alejandro Schcolnik-Cabrera, Alma Chavez-Blanco, Guadalupe Dominguez-Gomez, Mandy Juarez, Ariana Vargas-Castillo, Rafael Isaac Ponce-Toledo, Donna Lai, Sheng Hua, Armando R. Tovar, Nimbe Torres, Delia Perez-Montiel, Jose Diaz-Chavez, Alfonso Duenas-Gonzalez

**Affiliations:** 1 Division of Basic Research, National Cancer Institute, Ave. San Fernando 22, Tlalpan, 14080 Mexico City, Mexico; 2 PECEM, National Autonomous University of Mexico, Mexico City, Mexico; 3 Nutrition Physiology Department, National Institute of Medical Sciences and Nutrition, Salvador Zubiran, Mexico City, Mexico; 4 Division of Archaea Biology and Ecogenomics, Department of Ecogenomics and Systems Biology, University of Vienna, Vienna, Austria; 5 Molecular Biology Facility, University of Sydney, Sydney, Australia; 6 Pathology Department, National Cancer Institute, Mexico City, Mexico; 7 Unit of Biomedical Research in Cancer, Institute of Biomedical Research, National Autonomous University of Mexico, Mexico City, Mexico

**Keywords:** Cancer metabolism, Cancer therapy

## Abstract

The malignant energetic demands are satisfied through glycolysis, glutaminolysis and de novo synthesis of fatty acids, while the host curses with a state of catabolism and systemic inflammation. The concurrent inhibition of both, tumor anabolism and host catabolism, and their effect upon tumor growth and whole animal metabolism, have not been evaluated. We aimed to evaluate in colon cancer cells a combination of six agents directed to block the tumor anabolism (orlistat + lonidamine + DON) and the host catabolism (growth hormone + insulin + indomethacin). Treatment reduced cellular viability, clonogenic capacity and cell cycle progression. These effects were associated with decreased glycolysis and oxidative phosphorylation, leading to a quiescent energetic phenotype, and with an aberrant transcriptomic landscape showing dysregulation in multiple metabolic pathways. The in vivo evaluation revealed a significant tumor volume inhibition, without damage to normal tissues. The six-drug combination preserved lean tissue and decreased fat loss, while the energy expenditure got decreased. Finally, a reduction in gene expression associated with thermogenesis was observed. Our findings demonstrate that the simultaneous use of this six-drug combination has anticancer effects by inducing a quiescent energetic phenotype of cultured cancer cells. Besides, the treatment is well-tolerated in mice and reduces whole animal energetic expenditure and fat loss.

## Introduction

Cancer is characterized by cellular dysregulation, at the expense of high metabolic demands^1^. Like healthy cells, tumors obtain nutrients via biochemical pathways^2^. However, unlike healthy cells, which maintain a balance between anabolism and catabolism, malignant cells present a persistent anabolic state. Indeed, neoplastic cells increase the activity of the three main energetic pathways: glycolysis, glutaminolysis, and de novo fatty acid synthesis^3^. Tumors frequently over-express hexokinase-II (HK2), which processes glucose into pyruvate and lactate^4^. On the other hand, the systemic inflammatory environment in malignancy is associated with insulin resistance, lipolysis and proteolysis^5^. The negative nitrogen balance in the patient is accelerated by the tumor, which introduces glutamine released by the muscle, for processing with glutaminase (GLS) for energy generation, as well as for the synthesis of other amino acids, nucleotides and glutathione^6^. Additionally, with the overexpression of fatty acid synthase (FASN), cancer cells highly produce lipids for membrane biosynthesis, and as cholesterol and fatty acid deposits for energy and signaling^7^. This increased host catabolism in cancer patients associates with the cancer-associated cachexia syndrome^8^.

Although glycolysis, glutaminolysis and de novo fatty acid synthesis have been previously individually blocked^9–11^, there are no reports on the simultaneous blockade of tumor anabolism and host catabolism. Here we demonstrate the feasibility of a six-drug combination in mice. We blocked the tumor anabolism with orlistat, lonidamine and DON (6-Diazo-5-oxo-L-norleucine) (OLD drug scheme) to inhibit FASN, HK2 and GLS, respectively, whereas with the combination of anti-catabolic drugs we aimed to ameliorate cancer cachexia. Such combination (GII scheme) includes growth hormone (GH), insulin and indomethacin, which increases protein and lipid biosynthesis, stimulates glucose internalization, and reduces systemic inflammation, respectively^12–15^. This six-drug combination (OLD to inhibit tumor anabolism, plus GII to inhibit host catabolism) delays tumor growth in vivo without damaging normal tissues. Moreover, this treatment does not greatly affect mice metabolism as evaluated by VO_2_ consumption, while preserves global lean mass, despite a slight decrease in fat mass.

## Results

### The anti-anabolic compounds reduce cellular viability and clonogenicity, while induce cell cycle blockade and apoptosis

Our first question aimed to test cellular effects on the human colon adenocarcinoma cell line SW480, induced by the anti-anabolic (orlistat, lonidamine and DON) and anti-catabolic (GH, insulin and indomethacin) compounds, either alone or in combination.

**Figure 1:**
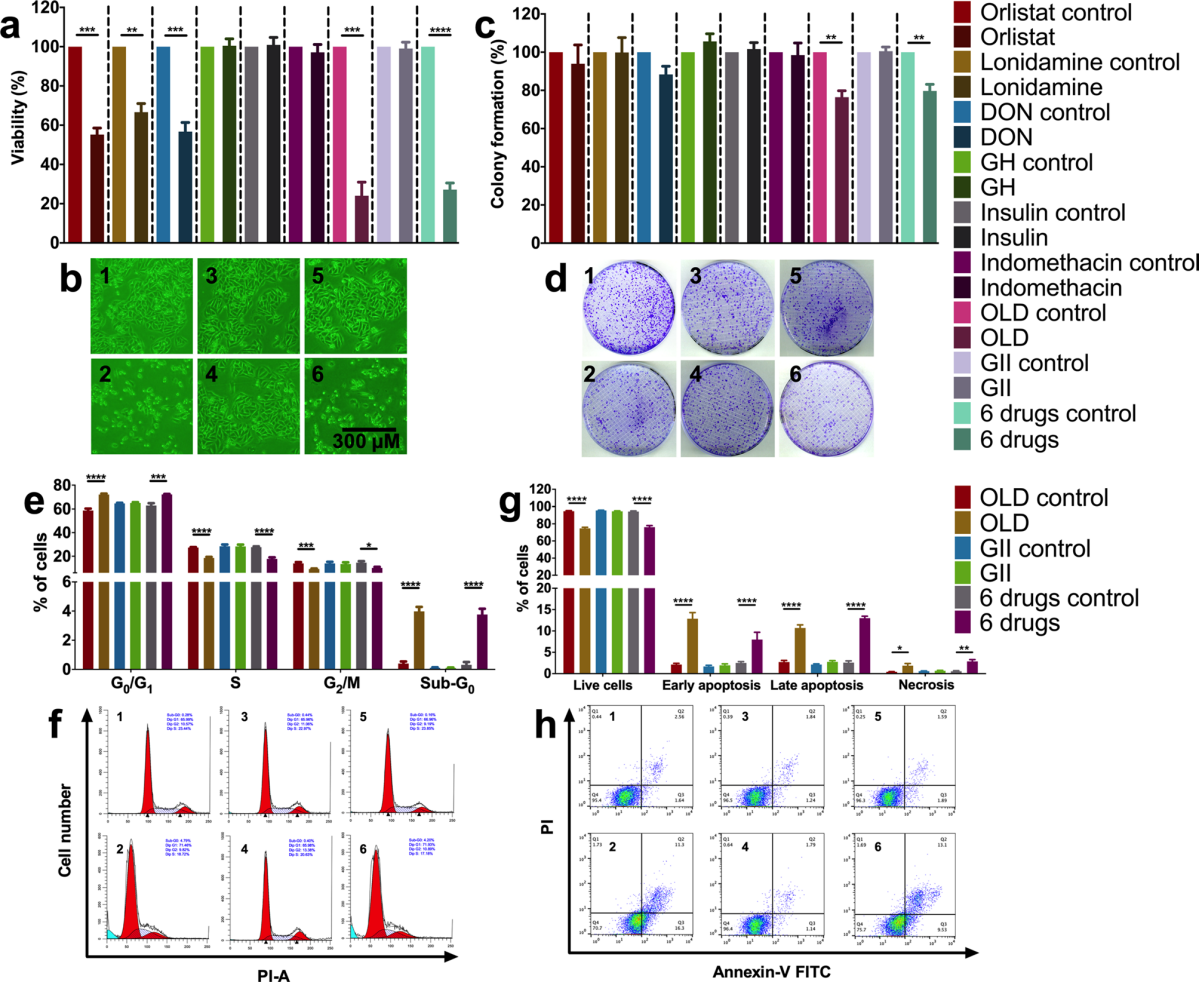
The blockade of the de novo synthesis of fatty acids, glycolysis and glutaminolysis reduces cellular viability and clonogenicity, and disrupts cell cycle with promotion of apoptosis. (**a**) Percentage of cellular viability. Values are normalized against its control. (**b**) Photography of cells after treatment with OLD control (1), OLD (2), GII control (3), GII (4), 6 drugs control (5), or 6 drugs (6). (**c**) Percentage of colony formation. Values are normalized against its control. (**d**) Scans of colonies at the end of the clonogenic assay, after treatment with OLD control (1), OLD (2), GII control (3), GII (4), 6 drugs control (5), or 6 drugs (6). (**e**) Cell cycle distribution. (**f**) ModFit diagrams after treatment with OLD control (1), OLD (2), GII control (3), GII (4), 6 drugs control (5), or 6 drugs (6). (**g**) Death distribution. (**h**) FlowJo diagrams showing alive (Q4), early apoptotic (Q3), late apoptotic (Q2), or necrotic (Q1). cells after treatment with OLD control (1), OLD (2), GII control (3), GII (4), 6 drugs control (5), or 6 drugs (6). Each condition was compared against its control. The images are representative of the data obtained. Data are expressed as means ± s.e.m. N = 3 independent experiments. Statistical analyses were performed with two-tailed unpaired Student t-test with Holm-Sidak correction. Scale bars = 300 μm. *GH: Growth hormone; OLD: Orlistat* + *lonidamine* + *DON; GII: GH* + *insulin* + *indomethacin; 6 drugs: OLD* + GII; *p < 0.05; **p < 0.01; ****p < 0.001; ****p < *0.0001*.

The individual use of orlistat, lonidamine and DON reduced around 50% cell viability. The employed concentrations of these drugs were the IC_40_, which previously proved to be synergistic^16^. No effect was seen with the individual or combined use of the anti-catabolic drugs (GII). On the contrary, a reduction of almost 80% was observed with either OLD or 6 drugs. A minor but statistically significant reduction was also seen for OLD and 6 drugs in clonogenicity (Fig. 1a–d). Flow cytometry evaluation using OLD and 6 drugs demonstrated a significant increase in G_0_/G_1_, and a decrease in S and G_2_/M cell cycle phases, while the proportion of cells in sub-G_1_ increased. No changes occurred with GII. Evaluation of cell death showed an increase with OLD and 6 drugs only (Fig. 1e–h).

### The anti-anabolic combinations change the expression of metabolic and cell cycle pathways

RNAseq was performed with SW480 treated with either the drug combinations or the control, composed of the vehicles of the six drugs. To this end, 34 h were selected to evaluate the effects during one SW480 replication cycle.

**Figure 2:**
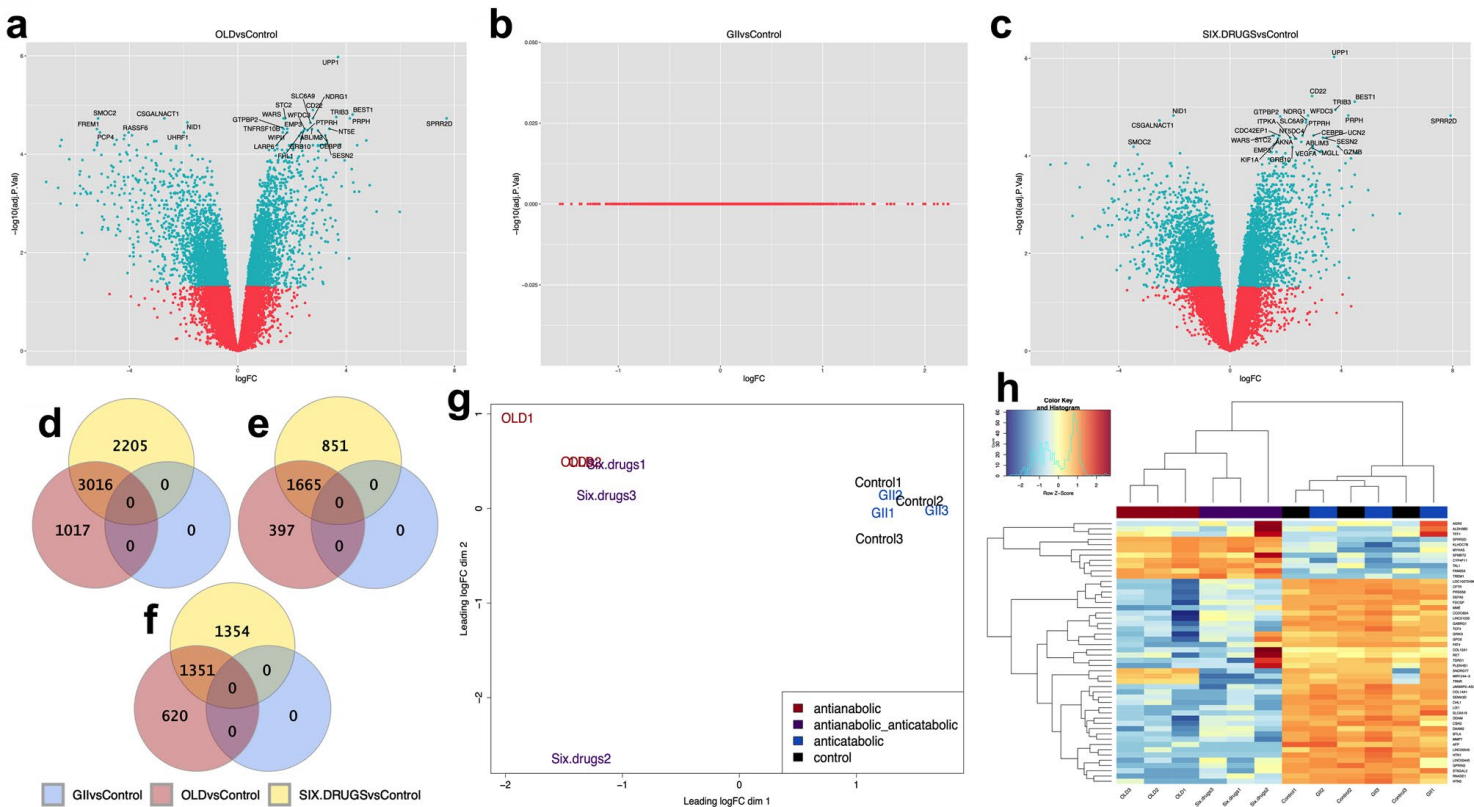
The metabolic treatment generates important changes on the transcriptomic profile. (**a-c**) Volcano plots of the OLD vs control (**a**), GII vs control (**b**) and 6 drugs vs control (**c**) conditions. Blue dots show statistically significant transcripts, and red dots show shared transcripts with no significant differences. The 30 most significant transcripts are indicated with their HUGO symbols in each plot. Blue dots located at the two upper-lateral quadrants indicate biologically and statistically differentially expressed transcripts. (**d-f**) Venn Diagram showing global differentially expressed genes (**d**), up-regulated genes (**e**) and down-regulated genes (**f**) in the OLD vs control, GII vs control, and 6 drugs vs control conditions. (**g**) Multidimensional scaling (MDS) plot of the 12 samples showing clustering between control and GII, as well as between most of the OLD and 6 drugs samples. (**h**) Hierarchical clustering with heatmap plot of the 50 most up-regulated transcripts identified in the 12 samples. Dendrograms showing a cluster between the anti-anabolic (OLD, red) and anti-anabolic + anti-catabolic (6 drugs, purple) samples, while the anti-catabolic (GII, blue) are mixed with the control samples (vehicles of the 6 drugs, black). N = 3 independent experiments. Statistical analyses were performed with one-way analysis of variance (ANOVA) with Bonferroni correction. *OLD: Orlistat* + *lonidamine* + *DON; GII: Growth hormone* + *insulin* + *indomethacin; 6 drugs: OLD* + *GII.*

Volcano plots demonstrated a similar pattern of expression changes in OLD vs. control and 6 drugs vs. control. Contrarily, no differential expression was observed in GII vs. control (Fig. 2a–c). Venn diagrams were constructed with the differentially expressed genes (DEGs) shared. OLD vs control had 1,017 DEGs, from which 397 were up-regulated and 620 down-regulated. 6 drugs vs control induced 2,205 DEGs, being 851 up-regulated and 1,354 down-regulated. OLD vs controls and 6 drugs vs control shared 3,016 DEGs, and no changes were found with GII vs control (Fig. 2d–f). The MDS plot revealed that all the GII and control samples clustered together, while most of the OLD and 6 drugs samples did cluster as well (Fig. 2g).

A heatmap was constructed with the most significant expressed genes, and it was confirmed that while OLD and 6 drugs clustered together, GII clustered with controls (Fig. 2h). Heatmaps analyzing the metabolism of glucose and glycogen, fatty acid synthesis and β-oxidation, and glutamine metabolism, were generated (Supplementary Fig. S1). OLD and 6 drugs downregulated *GLS1* and *HK2*, two targets of the drugs schemes, as well as *CPT1C*, a regulator of fatty acids transport into mitochondria for β-oxidation and of cancer cell senescence through metabolic reprogramming^17^.

The analysis with pathfindR led to identify and cluster the most significant altered pathways induced by OLD and 6 drugs (Supplementary Fig. S2). In both, the most important altered pathway was cell cycle. Additionally, pathfindR grouped the altered pathways in OLD (Supplementary Table S6) and 6 drugs (Supplementary Table S7), and showed the up- and down-regulated genes per pathway. For the purpose of this work, we are showing pathways related to cancer and metabolism. By using pathview in the 6 drugs vs control condition, we found multiple KEGG pathways with altered expression. Several genes of glycolysis/gluconeogenesis (Supplementary Fig. S3), oxidative phosphorylation (Supplementary Fig. S3), central carbon metabolism in cancer (Supplementary Fig. S4), and pathways in cancer (Supplementary Fig. S5), were altered. Finally, the next five genes were selected due to their high folding change in both OLD and 6 drugs conditions against their controls: upregulated, *CDK2B*, *FILIP1*, and *TNFSF14*; downregulated, *KIT* and *MMP7*. By RT-qPCR it was corroborated the same expression pattern in the referred genes, and interestingly, similar levels were seen between OLD and 6 drugs (Supplementary Fig. S6). Such results suggested important changes on cellular metabolism induced by the anti-anabolic drugs, and therefore we evaluated the energetic metabolism.

### The simultaneous metabolic blockade abolishes the energetic machinery, and restricts substrate flexibility

To investigate the effects of these drugs upon energetic metabolism, we quantified changes in oxidative phosphorylation, glycolysis, and fuel flexibility. We first conducted a time-course to identify the period in which the treatment started to induce metabolic changes. We found that 34 h of OLD produced similar and maintained effects as those found during the whole 72 h-period of treatment (data not shown), and therefore, 34 h was also selected for Seahorse assays.

**Figure 3:**
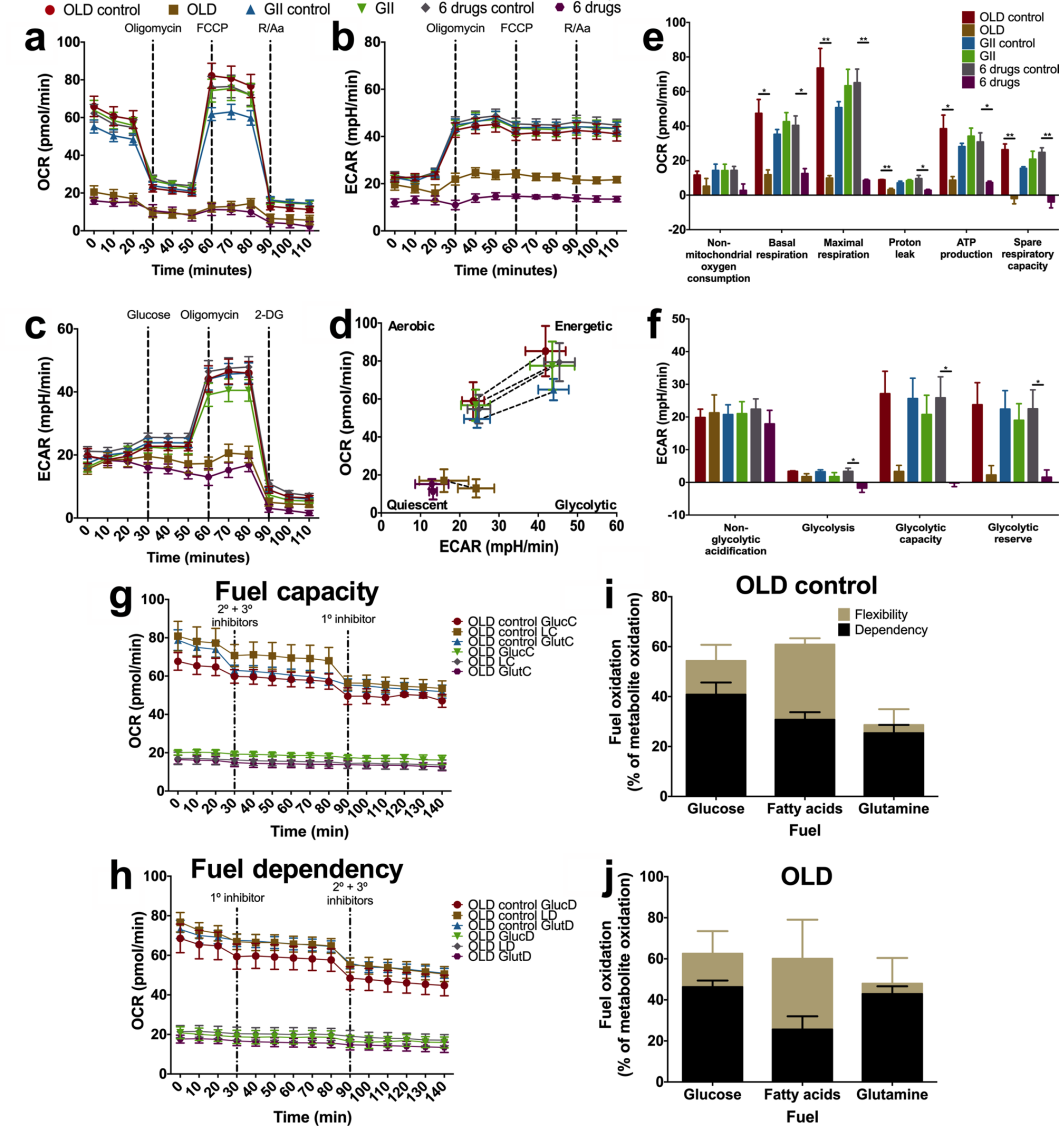
The triple energetic blockade alters the energetic machinery. Oxidative phosphorylation was evaluated by measuring OCR (**a**) and ECAR (**b**) prior to and after injecting oligomycin, FCCP, and a combination of rotenone (R) and antimycin A (Aa). (**c**) Glycolysis was evaluated by measuring ECAR prior to and after injecting glucose, oligomycin, and 2-deoxy-D-glucose (2-DG). (**d**) Energetic phenotype diagrams involving OCR and ECAR, under basal measurement and under the maximal stress after the injection of FCCP. (**e–f**) Individual parameters for oxidative phosphorylation (**e**) and for glycolysis (**f**). (**g-h**) Cellular respiration corresponding to substrate capacity (**g**) and dependency (**h**). (**i,j**) Fuel oxidation diagram representing the flexibility and dependency towards the three energetic substrates with OLD control (**i**) or OLD (**j**). The sum of flexibility and dependency indicates fuel capacity. Each condition was compared against its control. Data are expressed as means ± s.e.m. N = 3 independent experiments. Statistical analyses were performed with two-tailed unpaired Student t-test with Holm-Sidak correction. *OLD: Orlistat* + *lonidamine* + *DON; GII: Growth hormone* + *insulin* + *indomethacin; 6 drugs: OLD* + GII; GlucC: Glucose capacity; LC: Long-chain fatty acid capacity; GlutC: Glutamine capacity; GlucD: Glucose dependency; LD: Long-chain fatty acid dependency; GlutD: Glutamine Dependency; *p < 0.05; **p < 0.01*.*

OLD and 6 drugs led to a strong decrease in oxygen consumption rate (OCR) starting at basal measurements, as well as a reduction in extracellular acidification rate (ECAR), when the oxidative phosphorylation was evaluated (Fig. 3a,b). These effects resulted in almost a complete abolition of response after the addition of oxidative phosphorylation inhibitors. Therefore, several oxidative phosphorylation parameters were significantly inhibited (Fig. 3e). The glycolytic assay showed that OLD and 6 drugs decreased glycolysis, glycolytic capacity and glycolytic reserve (Fig. 3c,f). Finally, energetic phenotype charts generated with OCR and ECAR values at basal conditions and after the maximal stress with FCCP, indicated that OLD and 6 drugs severely impaired the energetic response, which induced a quiescent state (Fig. 3d). GII did not statistically modified any parameter.

Because OLD inhibits glycolysis, glutaminolysis and the de novo synthesis of fatty acids, we determined the “fuel” utilized by cells treated or not with OLD. The Seahorse XF Mito Fuel Flex Test reveals the cells' ability to switch oxidative pathways in meeting basal energetic demands, and provides information regarding the contributions of glucose, glutamine and long-chain fatty acid oxidation to basal respiration. As shown in Fig. 3g,h of fuel capacity and dependency, respectively, treatment with OLD led to almost fourfold less OCR production. As expected, no major modification resulted on the percentage of capacity to oxidize each of the fuels. Likewise, dependency was not major modified though there was a slightly increase in glucose and glutamine dependency, and a decrease in fatty acids dependency. Overall, there were no changes in the flexibility tendency. These data suggest a “frozen” state of the energetic phenotype by OLD (Fig. 3j), as compared against OLD control (Fig. 3i).

### The metabolic treatment impairs tumor growth, without affecting healthy tissues

**Figure 4:**
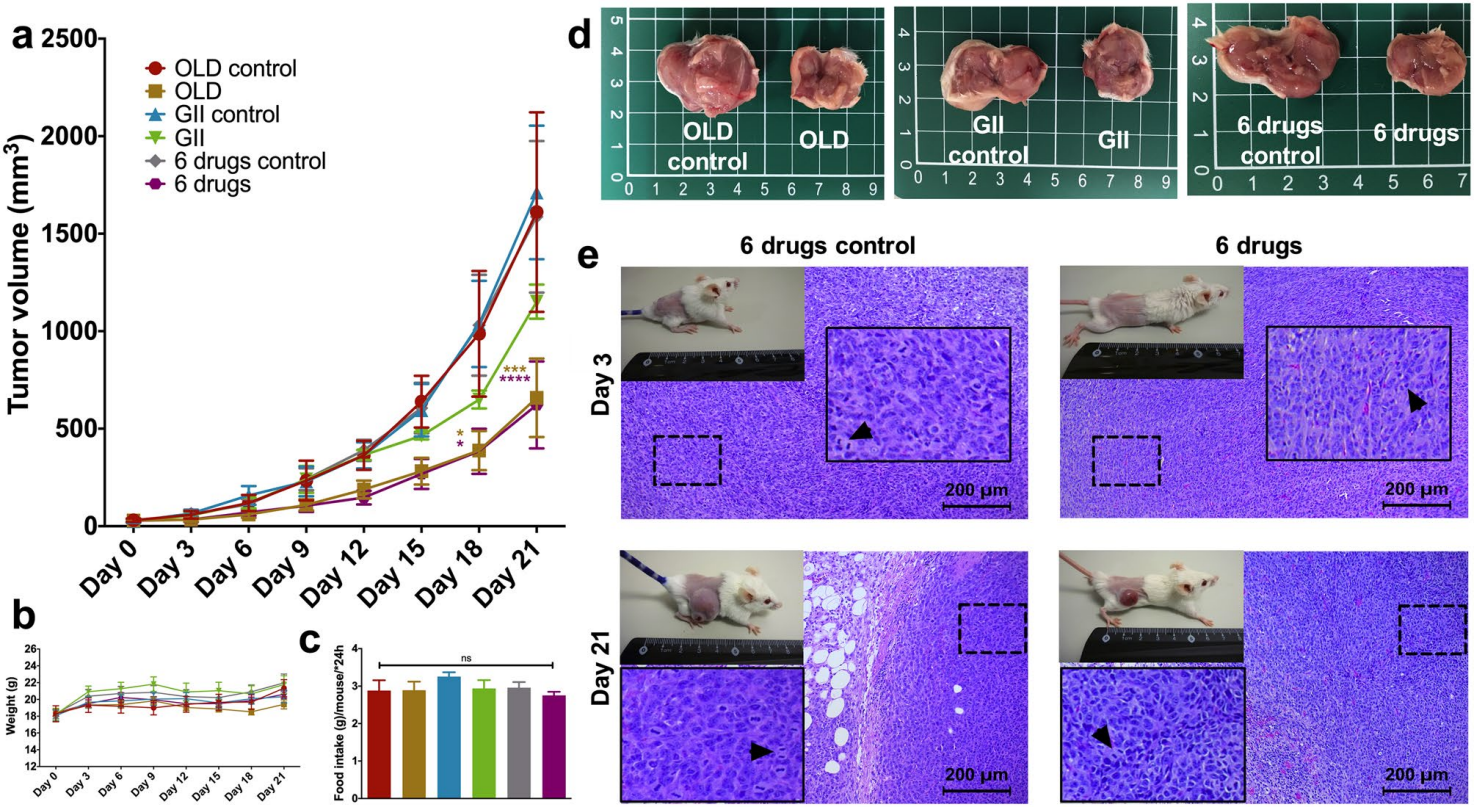
Tumor-bearing mice treated with the metabolic schemes have smaller tumor sizes. (**a**) 21-day time lapse of tumor volume with the OLD, GII, or 6 drugs combinations, or with their controls. (**b**) Weight changes over time. (**c**) Average food intake per mouse per 24 h. (**d**) Recovered tumor at day 21 of treatment. (**e**) Photography of mice at days 3 and 21 of treatment, and their respective H&E staining of isolated tumor samples, from the 6 drugs scheme. H&E Images are presented at 10X magnification, and a higher magnification shows with arrows examples of mitotic cells. Each condition was compared against its control. The images are representative of the data obtained. Data are expressed as means ± s.e.m. N = 3 independent experiments, 8 mice/group. Statistical analyses were performed with two-way ANOVA with Tukey correction. Scale bars = 200 μm. *OLD: Orlistat* + *lonidamine* + *DON; GII: Growth hormone* + *insulin* + *indomethacin; 6 drugs: OLD* + GII; ns: non-significant; *p < 0.05; ***p < 0.001; ****p < 0.0001*.*

Next, we evaluated tumor growth on CT26.WT-bearing mice treated with the metabolic combinations or their controls. At day 21, there was almost a threefold reduction in tumor volume with both OLD and 6 drugs. Interestingly, GII reduced the tumor volume though this difference was not statistically significant (Fig. 4a). No significant differences were found in mice weight (Fig. 4b) and food intake (Fig. 4c). Representative tumors at day 21 are shown in Fig. 4d, and H&E images depicting tumor slices from the 6 drugs groups at days 3 and 21 are shown in Fig. 4e. Of note, a reduction in mitosis was seen at day 3 of treatment with the 6 drugs condition (*p* < 0.05).

On Supplementary Fig. S7, photography of brain, lungs, heart, liver, colon, kidney, quadriceps muscle, brown fat, visceral fat, and subcutaneous fat from mice at day 21 of treatment are shown. There were no secondary changes in any of the mice groups. Furthermore, mice with no tumors and drug treatment had no evidence of tissue damage either (Supplementary Fig. S8).

### The 6 drugs scheme reduces oxygen consumption and limits fat loss

Since similar restrictive effects on tumor growth were seen between OLD and 6 drugs, we aimed to evaluate global metabolism in 6 drugs-treated mice. We measured oxygen consumption (VO_2_) at days 0 and 21 by indirect calorimetry. Along with the calorimetry assays, body composition by magnetic resonance imaging was performed. As controls of basal metabolism, healthy non-tumor bearing mice were treated.

**Figure 5:**
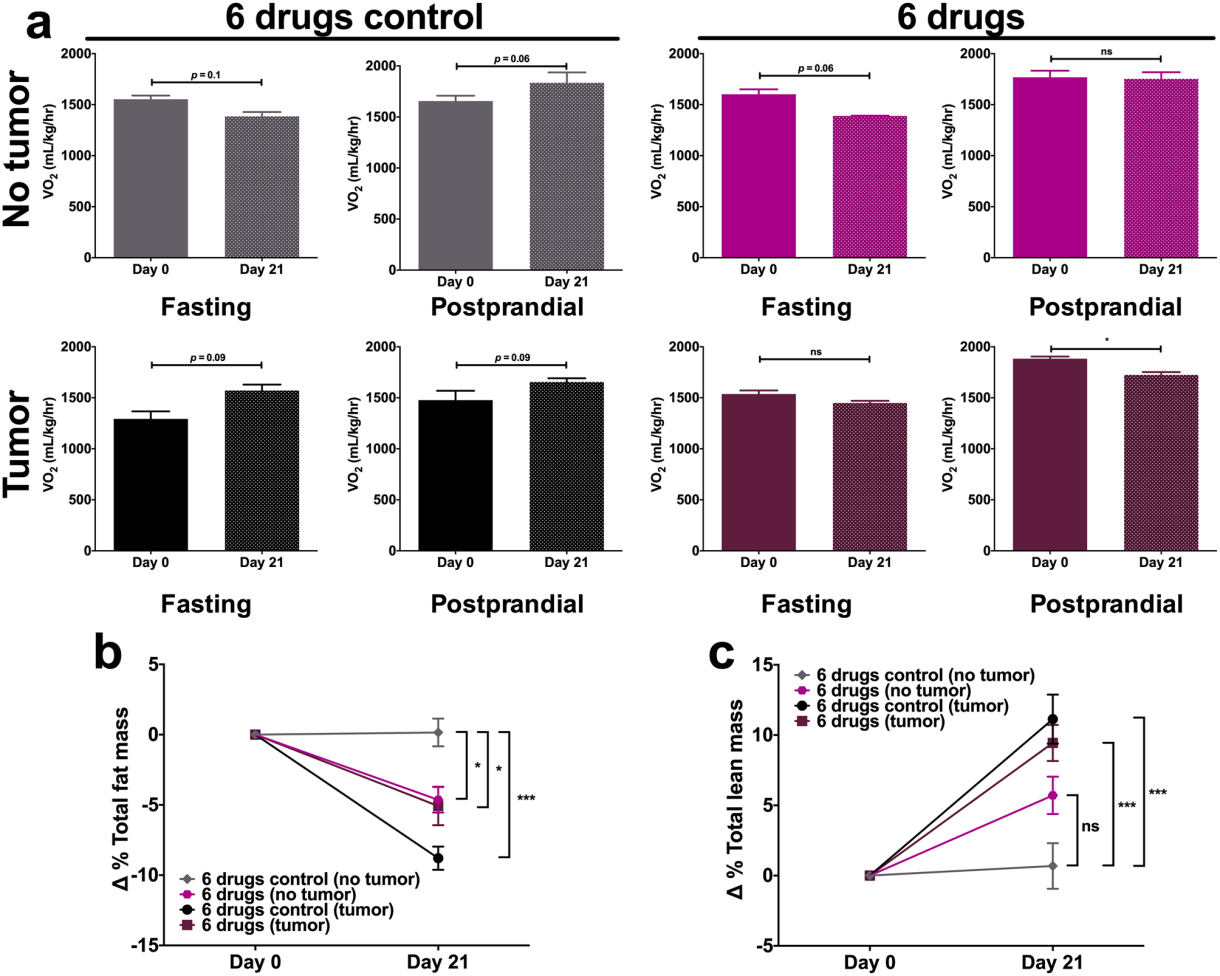
The chronic use of the 6 drugs combination reduces the energy expenditure and limits the loss of total fat mass in tumor-bearing mice. (**a**) Average oxygen consumption (VO_2_) values in the 6 drugs groups from non-tumor and tumor-bearing mice at days 0 and 21 of treatment, during a 24 h period composed of 12 h of fasting and 12 h of postprandial. (**b,c**) Delta of total fat (**b**) and lean (**c**) masses percentages at days 0 and 21 of evaluation*.* Data are expressed as means ± s.e.m. N = 1 independent experiment, 10 mice/group. Statistical analyses were performed with one-way ANOVA with Dunnet correction. *6 drugs: Orlistat* + *lonidamine* + *DON* + *growth hormone* + *insulin* + indomethacin; ns: non-significant; *p < 0.05; ***p < 0.001*.*

Results show no statistically significant differences in average VO_2_ in fasting and postprandial conditions in mice with no tumor, either treated or untreated. However, regarding control tumor-bearing mice, after 21 days of tumor growth the energy expenditure increased close to 21% in fasting conditions, while in the postprandial period such increase was higher than 12% (Fig. 5a). On the other hand, on tumor-bearing mice treated with the 6 drugs an opposite effect was seen, with a decrease of energy expenditure of 5.7% and 8.4% on fasting and postprandial periods after 21 days of treatment, respectively, being the difference significant in the latter.

Regarding body composition, total fat mass reduced over time in mice with no tumor and treated, and a comparable effect was seen in tumor-bearing mice and treated. However, control mice with tumor demonstrated a higher fat loss (Fig. 5b). Both groups with tumor increased their percentage of total lean mass, although control mice had higher percentage of lean mass (Fig. 5c). No significant differences were seen in lean mass between mice without tumor.

### The 6 drugs scheme in tumor-bearing mice normalizes the glucose tolerance curve and reduces Ucp1 expression in subcutaneous fat

**Figure 6:**
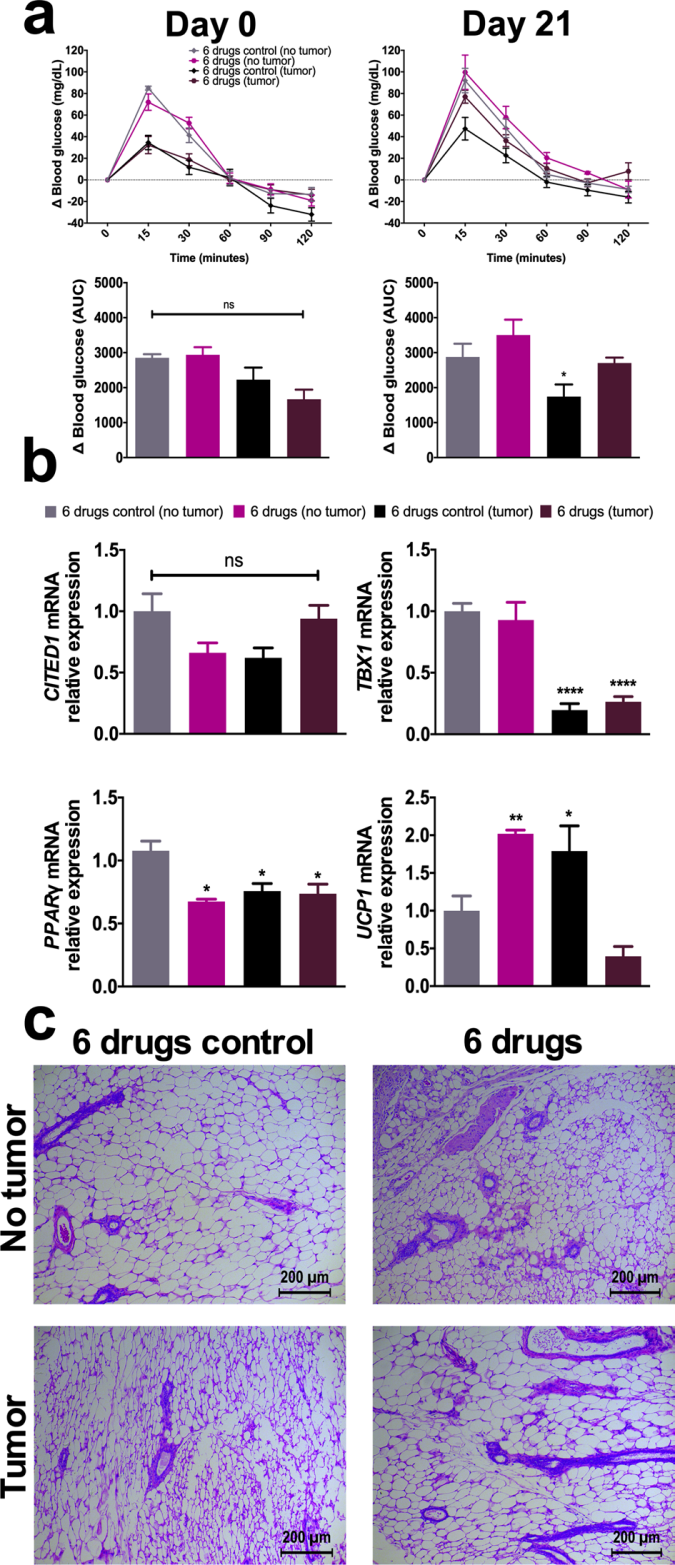
The 6 drugs scheme reverts the low glucose peak seen in non-treated, tumor-bearing mice, and prevents the loss of subcutaneous fat mass. (**a**) Delta of blood glucose at days 0 and 21 of treatment, and the area under the curve per condition. (**b**) mRNA relative expression of *CITED1*, *TBX1*, *PPARγ*, and *UCP1*. (**c**) H&E staining of subcutaneous fat samples isolated after 21 days of treatment. H&E Images are presented at 10X magnification. The images are representative of the data obtained. Data are expressed as means ± s.e.m. N = 1 independent experiment, 10 mice/group. Statistical analyses were performed with one-way ANOVA with Dunnet correction. Scale bars = 200 μm. *6 drugs: Orlistat* + *lonidamine* + *DON* + *growth hormone* + *insulin* + *indomethacin*; AUC: Area under the curve; ns: non-significant; *p < 0.05; **p < 0.01; ****p < 0.0001.

On days 0 and 21, glucose tolerance tests were performed on healthy and tumor-bearing mice. At day 0, mice with tumor had lower glucose peaks 15 and 30 min after the glucose solution injection, as compared with their healthy counterparts. Regarding the 6 drugs scheme, there were no differences in the area under the curve (AUC) of delta blood glucose at day 0. However, at day 21, a significant decrease was observed in untreated tumor-bearing mice, while treated mice with tumor demonstrated similar glucose concentrations in blood to those seen in mice without tumor (Fig. 6a).

Finally, since we detected that tumor-bearing mice had a tendency to “engulf” ipsilateral inguinal subcutaneous fat as the tumor grew, total RNA was recovered from such area to elucidate changes in the expression of molecules related with adipogenesis and fat transition from white to brown. As a comparison, subcutaneous fat from mice without tumor recovered from the same region to that in tumor-bearing mice was employed. Figure 6b shows decrease in *PPARγ*, in all but untreated mice without tumor; the beige adipocyte marker *TBX1* decreased in both, untreated and treated with tumor; and *UCP1*, indicative of thermogenesis, increased in the untreated mice with tumor, and in the treated mice without tumor. Though non-statistical significant, treated mice with tumors had a trend for decreasing this marker. Figure 6c displays representative H&E staining of subcutaneous fat of each evaluated group. Multilocular adipocytes in white fat are seen in untreated mice with tumor, and in treated mice without tumor.

## Discussion

Our results show that the simultaneous inhibition of glycolysis, glutaminolysis and de novo synthesis of fatty acids with OLD or the six-drug combination, leads to in vivo and in vitro antitumor effects. Both combinations induce a strong inhibition of mitochondrial function; significant changes in whole transcriptome; and most importantly, no undesirable clinical or pathological changes in mice. The six-drug combination reduces energetic expenditure induced by the tumor; ameliorates fat mass loss percentage with no changes in lean mass, and “normalizes” glucose levels in tumor-bearing mice.

The realization that metabolic alterations, specifically, higher rates of glycolysis, glutaminolysis and de novo synthesis of fatty acids, are second-generation cancer hallmarks, has opened the door for targeting the altered metabolism in cancer as a newer form of cancer therapy^18–20^. Nonetheless, so far no metabolic drug aiming any of these alterations has been FDA-approved, though novel chemical entities, targeting critical enzymes for these metabolic routes, are in preclinical and early-phase clinical studies^21^.

Here we show that by employing two drugs clinically tested in the 80s, which are inhibitors of glycolysis –lonidamine-^22,23^ and glutaminolysis –DON-^24–26^, plus the well-known inhibitor of FASN -orlistat-^10,21^, effective in vitro and in vivo effects against colon cancer cells are found. This combination, termed ‘OLD’, is aimed to “block” tumor anabolism^19,20^. On the other hand, cancer cachexia is considered as a catabolic state of the host induced by the tumor itself^8,27^. On this basis, here we added GH, insulin and indomethacin in the GII scheme. These drugs increase protein biosynthesis, stimulate glucose internalization, increase lipogenesis, and reduce systemic inflammation^12–15^.

Confirming our previous findings^16^, we observed strong growth inhibitory effects in vitro which were associated with G_0_/G_1_ arrest of cell cycle and increased apoptosis. As far as we know, there are no publications testing cell cycle or cell death with a combination of inhibitors of these pathways. A publication that assessed in leukemia cells a lonidamine + DON combination, observed an increased anti-leukemia effect when both inhibitors are used together^28^, and similar effects were observed in lung cancer cells, with the combination of lonidamine and the glutaminase inhibitor 968^29^. Regarding the GII combination, despite that GH raises concerns about its pro-tumoral effects^30^, in vitro and in vivo studies demonstrate that it does not stimulate cancer progression^31–33^. Insulin, on the other hand, does increase malignant cell proliferation and invasiveness^34,35^, but these effects are at least inconsistent regarding cancer risk and survival in patients^36–38^. According with these studies, in our model neither GH, insulin, nor indomethacin modified cancer growth in vitro or in vivo. This occurred despite we confirmed by RT-qPCR (data not shown) that SW480 colon cancer cells do express GH and insulin receptors, as well as cyclooxygenase. Hence, the lack of pro-tumoral effects cannot be attributed to null expression of these molecules.

Interestingly, OLD and 6 drugs, but no GII, induced a significant decrease in oxidative phosphorylation, and glycolysis as well (6 drugs only). Overall, the results indicated that both OLD and 6 drugs severely impaired the energetic response in treated cells. Essentially, treated cells “freeze” at basal conditions, while the controls and GII-treated cells did respond to stress by increasing OCR and ECAR, indicative of mitochondrial respiration and glycolysis, respectively. Comparatively, the extent of OCR was as high as that achieved with the mitochondrial targeting compound Dodecyl-TPP in breast cancer cells^39^, and the extent of glycolysis inhibition as observed in lonidamine-treated lung cancer cells^29^. Furthermore, we found that OLD does not greatly modify the total capacity of cells to oxidize glucose, fatty acids and glutamine, though a small decrease in fatty acid and glutamine oxidation dependency was observed. These minor changes are expected as full enzyme inhibition could not be guaranteed at the micromolar concentrations here used. Nevertheless, the “frozen” energetic phenotype observed well-corresponds to absence of flexibility changes in these fuels, and more importantly, the absolute OCR production was inhibited with OLD at basal conditions and remained so after adding glycolysis, glutaminolysis and β-oxidation inhibitors, which “force” cells to use alternative fuels.

So far, there is no information on the whole-genome transcriptional response to combined treatment with inhibitors of glycolysis, glutaminolysis and de novo synthesis of fatty acids. Here we show that OLD and 6 drugs induce almost an identical transcriptional response, while untreated controls and GII alone did not change the transcriptional landscape. This confirms that GII has no or minor effects in colon cancer cells, and as expected, OLD and 6 drugs modulate the expression of, among others, glycolysis/gluconeogenesis, oxidative phosphorylation, central carbon metabolism, glutamine, lipid metabolism, and both de novo synthesis and β-oxidation of fatty acids. We did not validate at protein level and did not assess the enzymatic activities of these inhibitors. Therefore, we cannot state the overall effect upon pathway activity of the combinations. However, on the basis of the inhibition of glycolysis, oxidative phosphorylation and absolute decrease in OCR and unchanged flexibility, we can suggest that the transcriptional changes in metabolic pathways were unfavorable to tumor growth.

The in vivo assays of syngeneic tumor graft in mice showed that the anti-anabolic treatments were effective in reducing tumor growth. Unexpectedly, GII had a non-significant but clear effect in tumor reduction, confirming the absence of tumorigenic effect of this combination. The fact that the treatment did not induce changes in food intake, animal weight and observable clinical signs of toxicity, argues in favor that, somehow, the three anabolic inhibitors do exhibit certain level of specificity toward cancer cells, as normal cells transiently increase the activity of these pathways when they are on high metabolic demands^40^. Moreover, it is unlikely that the enzymatic activity of HK2, GLS and FASN were completely shut-down, because of pharmacodynamics and pharmacokinetic issues. From this perspective, it is reasonable to expect that the metabolic blockade was enough to achieve an antitumor response, without greatly affecting the functioning of normal cells. In fact, the six-drug treatment seems to have a favorable effect as the energetic expenditure, as measured by VO_2_, was decreased in tumor-bearing animals. Increased resting VO_2_ is observed upon TNF and IL-1β injection in vivo^41–43^, and these cytokines are known to be produced by tumors^8,41^.

Regarding changes in body composition, fat loss is an early finding in cancer cachexia, which is attributed to increased lipolytic pathways and browning of white fat mass, conferring thermogenic properties of adipocytes and wasteful energy expenditure^41–44^. The minor percentage of fat mass here observed with the six-drug treatment could have resulted from either less adipogenesis, as *PPARγ* mRNA decreased in all groups but the untreated mice without tumor, or/and by a potential increase in thermogenesis, as animal with tumor and untreated, and animals without tumor and treated, had higher *UCP1* expression. On the other hand, the data obtained with *TBX1* and *CITED1* expression suggests absence of beige differentiation in subcutaneous fat^44^. However, further evaluation is needed to understand the lipolytic effect related with tumorigenesis and treatment.

In summary, this study demonstrates that in this experimental model, the triple anabolic blockade does not only induce antitumor effects and greatly affects the energetic machinery of colon cancer cells, but is well-tolerated, without whole animal toxic effects. Moreover, this combination induces a transcriptional effect of predicted metabolic pathways intended to be targeted. Whether GII ameliorates cancer cachexia remains to be demonstrated, as no cachexia was observed in untreated tumor-bearing mice. However, the changes on VO_2_ and amelioration in fat loss percentage suggest that it could have an anti-cachectic effect. This work uncovers the feasibility of targeting both cancer-related phenomena, tumor anabolism and host catabolism. Hence, this concept must be further explored.

## Methods

### Ethical statement

All animal experiments were approved by the Ethics and Scientific committees of the National Cancer Institute (protocol numbers 017/009/IBI and CEI/1055/17), and by the Animal Committee of the National Institute of Medical Sciences and Nutrition Salvador Zubiran (protocol number FNU-1927-18-19-1), both from Mexico City, Mexico. All experiments were performed in accordance with relevant guidelines and regulations.

### Cell culture

The human and mouse colon adenocarcinoma cell lines SW480 and CT26.WT (ATCC), respectively, were employed. Cells were plated in DMEM/F12 or RPMI-1640 (both from Gibco), for SW480 and CT26.WT, respectively, supplemented with 10% fetal bovine serum (Corning) and 1% streptomycin/amphotericin (Gibco) (complete medium), at 37 ºC in a 5% CO_2_ incubator.

### Drugs

Orlistat (Psicofarma), lonidamine (Sigma), 6-Diazo-5-oxo-L-norleucine (DON) (Sigma), growth hormone (GH) (Merck), insulin (Lilly), and indomethacin (Sigma) were employed. Orlistat and indomethacin were dissolved in absolute ethanol (Sigma), lonidamine in DMSO (Sigma), and DON, GH and insulin in complete medium. The compounds were administered alone or in the anti-anabolic (orlistat + lonidamine + DON, named ‘OLD’), anti-catabolic (GH + insulin + indomethacin, named ‘GII’), or 6 drugs (OLD + GII, named ‘6 drugs’) schemes.

### Cell viability and clonogenicity

5 × 10^4^ SW480 cells/well were seeded in 6-well plates (Costar), with 2 mL of complete medium. After 24 h of pre-incubation, cells were treated during 72 h with orlistat, lonidamine, or DON at synergistic concentrations, as stated before^16^, and with the maximum circulating concentrations of GH, insulin, or indomethacin, reported in healthy subjects^45–47^. The concentrations are found on Supplementary Table S1. Briefly, the doses were: orlistat, 8.7 μM; lonidamine, 75.86 μM; DON, 6.12 μM; GH, 0.87 nM; insulin, 0.809 nM; indomethacin, 7.5 μM. 9 conditions were evaluated: Each drug alone, and the OLD, GII and 6 drugs combinations. Each condition was compared against its control, composed by the vehicle(s) at the same volume. Fresh complete medium containing each drug/vehicle was changed every 24 h. After 72 h, cells were counted as previously described^48^. Next, 1000 cells/condition were recovered and plated in new 6-well plates for clonogenic assays, following Dominguez-Gomez et al.^49^, and were counted with ImageJ V2.0 (NIH, MA, USA).

### Cell cycle, apoptosis and necrosis

5 × 10^4^ SW480 cells/well treated with the combinations or their controls, as indicated above. Then, cells were recovered and dyed with propidium iodide (Sigma) during 1 h, and 20,000 events/sample were analyzed with the BD FACSCanto II flow cytometer (BD Biosciences). Cell cycle analysis was performed with ModFit LT V2.0 (Verity Software House). In independent assays, after 72 h of treatment with the combinations or their controls, cells were recovered and dyed with annexin-V and propidium iodide, with the Annexin-V-FLUOS Staining Kit (Roche). Apoptosis and necrosis were simultaneously collected with 10,000 events/sample by flow cytometry, and the data was analyzed with FlowJo V10.7.1 (Becton Dickinson & Company, USA).

### RNA preparation and sequencing

5 × 10^4^ SW480 cells/well were treated during 34 h with the OLD, GII, or 6 drugs schemes, as stated above. The vehicles of the six drugs were used as controls. Next, total RNA was extracted with TRIzol (Invitrogen, Carlsbad, CA, USA) by following the manufacturer’s instructions. Library conversion was performed with poly-A selection with the Illumina TruSeq Stranded mRNA library preparation kit LT (Illumina), by following the manufacturer’s instructions. Libraries were pooled prior to sequencing. Samples were sequenced on the Illumina HiSeq 2500 with V4.0 chemistry, generating 2- by 125-bp paired-end reads. For a further explanation of RNA preparation and sequencing, and for the detailed description of quality control, mapping, differential expression analysis, and gene ontology and pathway enrichment analysis, please refer to Supplementary Data and to^50–58^. The methodology followed for RT-qPCR for validation of selected genes is also found in Supplementary Data. The complete set of primers employed is listed in Supplementary Table S2.

### Oxidative phosphorylation with the XF Cell Mito Stress Test and glycolysis with the XF Glycolysis Stress Test

For the information regarding cellular preparation for extracellular flux analysis, please refer to Supplementary Data. The experimental design of both oxidative phosphorylation and glycolysis assays followed the stated by Zaytseva et al*.*^59^. OCR (pmoles/min) and ECAR (mpH/min) were measured for oxidative phosphorylation, and only ECAR was considered for glycolysis. 12 measurements/assay were made.

### Fuel flexibility with the XF Mito Fuel Test

We followed the manufacturer’s protocol, and only considered OCR. The injection order of each inhibitor/pair of inhibitors, required to evaluate capacity, dependency and energetic flexibility, are found on Supplementary Table S3. 15 measurements/assay were made.

### Mice tumor growth

This study was carried out in compliance with the ARRIVE guidelines. All mice were maintained at the animal facilities in the National Cancer Institute and in the National Institute of Medical Sciences Salvador Zubiran (Mexico City, Mexico). Eight 7–9 weeks old female Balb/c mice/group, 18.13 ± 1.66 g, were employed per replicate. 2–3 mice/cage were maintained in 12 h illumination/12 h darkness at 22 ± 2 ºC, with adequate ventilation, and cleaning of cages twice per week. Water and AIN-93G diet were administered ad libitum. After a 7 day-period of acclimation, 5 × 10^5^ CT26.WT cells resuspended in 100 μL 0.9% sterile saline solution were subcutaneously injected in one flank/mouse. Cells had a passage number < 10, and cellular viability ≥ 95%. After identifying with a caliper a tumor size ≥ 3 mm. in major axis, mice were randomized considering weight and tumor volume to ensure homogeneity. Tumor volume was calculated according to Shaw R. et al., with the formula Volume = (Major axis*minor axis^**2**^)*(π/6)^60^. The drug schemes and doses are found on Supplementary Table S5, and are based on previous results from our group^61^ and Chen ^62^. Each condition was intraperitoneally administered and compared against its control, composed of the vehicles at the same volumes. Mice were clinically evaluated every third day, and were sacrificed by cervical dislocation following the Mexican regulations for animal handling NOM-062-ZOO-1999.

### Pathologic analysis

2 mice/group were randomly sacrificed on day 3 of treatment, and all others at day 21. Tissue samples were immediately fixed in a 10% formaldehyde solution. Each tissue was embedded in paraffin, cut into 5 μm slices, and stained with hematoxylin and eosin (H&E). The samples were blindly analyzed by a pathologist with a microscope (Leica DM750, Wetzlar, Germany), photographed with a digital camera (Leica DMC2900), and processed with Leica LAS Core V4.5.

### Indirect calorimetry

Energy expenditure was quantified by indirect calorimetry in independent experiments with 10 mice/group, under the same conditions as indicated above. Briefly, calorimetric chamber allows to analyze the flux rate with a mass flux controller. On days 0 and 21, oxygen consumption (VO_2_, mL/kg/h) was quantified every 90 s, to calculate the energetic expenditure. The VO_2_ was individually monitored in plexiglass chambers with an open flux system connected to an Oxymax-CLAMS (CLAMS, Columbus Instruments, OH) system during 24 h, composed by an initial 12 h-period of fasting (7:00–19:00 h, light cycle), followed by 12 h of food ad libitum (19:00–7:00 h, dark cycle). All animals underwent a 12 h-acclimatizing period prior the beginning of the assay.

### Body composition

Lean and fat mass were measured with quantitative magnetic resonance imaging (EchoMRI, Echo Medical Systems, Houston, TX, U.S.A.) on days 0 and 21, previously to calorimetric assays. Scans were performed as previously described^63^. Fat and lean mass compositions were expressed as delta percentages against basal measurements at day 0.

### Intraperitoneal glucose tolerance tests

On days 0 and 21, and before body composition experiments, an intraperitoneal injection of a 2 g glucose/kg body weight solution was performed per mouse, under an 8 h-period of fasting. Tail-nick blood samples and glucose measurements were prepared as previously described^63^. Glucose measurements were expressed as delta blood glucose concentrations against basal quantification at time 0.

### Quantitative real-time PCR of subcutaneous fat

Total RNA was extracted from the subcutaneous inguinal fat with TRIzol reagent, by following the manufacturer’s instructions. When the mouse had tumor, fat was recovered from the ipsilateral inguinal region relative to the neoplasia. RNA (3000 ng) was converted to cDNA through reverse transcription. Quantitative real-time polymerase chain reaction was conducted as described before^63^ in order to assess the expression of *CITED1*, *TBX1*, *PPARγ*, and *UCP1*. The primers sequences are found on Supplementary Table S4.

### Statistical analysis

Unless otherwise specified, all experiments were independently performed in triplicate, with three internal replicates. Statistical analyses were performed as follows: Two-tailed unpaired Student t-test with Holm-Sidak correction for cellular viability, clonogenicity, cell cycle, apoptosis and Seahorse assays; one-way analysis of variance (ANOVA) with Bonferroni correction for RNAseq data; two-way ANOVA with Tukey correction for tumor size comparison; and one-way ANOVA with Dunnet correction for delta comparison of total fat mass, total lean mass, delta of area under the curve of delta blood glucose, and mRNA relative expression of adipose and SW480 transcripts. The results were analyzed with GraphPad Prism V6 (GraphPad, San Diego, CA, USA). Data were expressed as means ± s.e.m. *p* < 0.05 was considered statistically significant.

## Supplementary Information

Supplementary Information

## Data Availability

The datasets are available from the corresponding author on reasonable request.

## Abbreviations

HK2: Hexokinase-II
GLS: Glutaminase
FASN: Fatty acid synthase
DON: 6-Diazo-5-oxo-L-norleucine
OLD: Orlistat + lonidamine + DON
GH: Growth hormone
GII: GH + insulin + indomethacin
6 drugs: OLD + GII
VO_2_: Oxygen consumption
DEGs: Differentially expressed genes
MDS: Multidimensional scaling
CPT1C: Carnitine palmitoyltransferase 1C
OCR: Oxygen consumption rate
ECAR: Extracellular acidification rate
FCCP: Carbonyl cyanide-4-(trifluoromethoxy)phenylhydrazone
H&E: Hematoxylin and eosin
AUC: Area under the curve
PPARɣ: Peroxisome proliferator-activated receptor gamma
TBX1: T-box transcription factor 1
UCP1: Uncoupling protein 1
CITED1: Cbp/P300 interacting transactivator with glu/asp rich carboxy-terminal domain 1
CDKN2B: Cyclin dependent kinase inhibitor 2B
FILIP1: Filamin A interacting protein 1
TNFSF14: TNF superfamily member 14
KIT: KIT proto-oncogene, receptor tyrosine kinase
MMP7: Metalloproteinase 7
