# Supplementary material for: Pharmacological inhibition of tumor anabolism and host catabolism as a cancer therapy
Source: Sci Rep. 2021 Mar 4;11:5222. doi: 10.1038/s41598-021-84538-6 (PMC7933231; doi:10.1038/s41598-021-84538-6)
Supplement: Supplementary file 1 — Supplementary Information [file 41598_2021_84538_MOESM1_ESM.docx]

**Pharmacological inhibition of tumor anabolism and host catabolism as a cancer therapy**

Alejandro Schcolnik-Cabrera^1,2^, Alma Chavez-Blanco^1^, Guadalupe Dominguez-Gomez^1^, Mandy Juarez^1^, Ariana Vargas-Castillo^3^, Rafael Isaac Ponce-Toledo^4^, Donna Lai^5^, Sheng Hua^5^, Armando R. Tovar^3^, Nimbe Torres^3^, Delia Perez-Montiel^6^, Jose Diaz-Chavez^1^, Alfonso Duenas-Gonzalez^1,7^*

^1^Division of Basic Research, National Cancer Institute, Mexico City, Mexico.

^2^PECEM, National Autonomous University of Mexico, Mexico City, Mexico.

^3^Nutrition Physiology Department, National Institute of Medical Sciences and Nutrition, Salvador Zubiran, Mexico City, Mexico.

^4^Division of Archaea Biology and Ecogenomics, Department of Ecogenomics and Systems Biology, University of Vienna, Vienna, Austria.

^5^Molecular Biology Facility, University of Sydney, Sydney, Australia.

^6^Pathology Department, National Cancer Institute, Mexico City, Mexico.

^7^Unit of Biomedical Research in Cancer, Institute of Biomedical Research, National Autonomous University of Mexico, Mexico City, Mexico.

**Supplementary Methods**

*RNA preparation and sequencing*

RNA was quantified at A260, and its quality was analyzed at A230/260 and A260/280 ratios (QIAxpert, Qiagen, Hilden, Germany). RNA integrity (RIN) was evaluated with RNA 6000 Nanochips on a 2100 Bioanalyzer equipment (Agilent). Samples with a RIN value >9 were considered. Transcriptomic assays took place at the Kinghorn Centre for Clinical Genomics in Sydney, Australia. Casava V8.2 (Illumina) was employed for base calling and demultiplexing of indexed reads, to convert data in fastq format.

*Quality control and reads mapping*

The raw fastq files were trimmed with Trimmomatic to remove Illumina adapter sequences^1^. The quality of the trimmed reads was evaluated with FastQC. Paired-end FASTQ files were combined from lane-level files for samples, and reads were mapped to the human reference genome (GRCh38) using Rsubread^2^. The number of mapped reads/gene were counted with featureCounts.

*Differential expression analysis*

Raw counts were normalized according to library size to obtain counts-per-millions (cpm) with edgeR^3^. Genes with cpm<0.5 and present in <3 samples (control and treatments had each three biological replicates) were removed. The filtered dataset was mapped to Entrez Gene identifiers using the genome wide annotation org.Hs.eg.db. Differential expression was analyzed with voom function from limma^4^ according to a paired design (control vs treatments). False discovery rates (FDRs) <0.05 were considered as differentially expressed genes (DEGs).

*Gene Ontology and Pathway Enrichment analyses*

Gene Ontology enrichment was tested for over-representation in DEGs with the goana function from limma^4^, with FDR cutoff at 0.05 for multiple testing. KEGG^5-7^ pathway enrichments were tested with pathfindR via identification of active-subnetworks of DEGs (adjusted *p*<0.05)^8^, and those pathways were visualized with pathview^9^.

*Expression of genes by reverse transcription-quantitative polymerase chain reaction (RT-qPCR)*

5x105 SW480 cells were seeded in 6-well plates, and were allowed to attach overnight for drug treatment. Cells were treated during 34h with either the OLD scheme or with its control, or with the 6 drugs or its control. After that period, cells were washed once with 1X PBS and were recovered with a 0.05% trypsin-0.02% EDTA solution (Sigma Aldrich, Missouri, USA). Total RNA isolation was carried out using TRIzol (Invitrogen Life Technologies, Carlsbad, CA, USA), by following the manufacturer’s instructions.

RNA purity and integrity were assessed by spectrophotometric analysis using a NanoDrop 2000c (Thermo Fisher Scientific, Inc.) and denaturing 2% agarose gel; bands were visualized using a MiniBIS Pro D-Transilluminator (DNR Bio-Imaging Systems Ltd., Neve Yamin, Israel). A total of 1 µg total RNA was used for cDNA synthesis with the GeneAmp RNA PCR Core kit (Applied Biosystems; Thermo Fisher Scientific, Inc.). cDNA was used with iQ SYBR Green SuperMix (Bio-Rad Laboratories, Inc.), according to the manufacturer's protocol. qPCR reactions were run in triplicate using an ABI Prism 7000 equipment (Applied Biosystems; Thermo Fisher Scientific, Inc.). The qPCR cycling conditions were as follows: 10 min at 95˚C; 40 cycles of 30 sec at 95˚C; 30 sec at 60˚C, and 30 sec at 72˚C. Data were analyzed using the 2-ΔΔCq method, and reported as the fold-change in gene expression normalized to the endogenous control gene hypoxanthine phosphoribosyltransferase 1 (*HPRT1*), and relative to cells without treatment. The complete list of employed primers is found in **Supplementary Table S2**.

*Extracellular flux analysis*

Glycolysis, oxidative phosphorylation and fuel flexibility were evaluated with the Seahorse Bioscience Extracellular Flux Analyzer XF96e (Seahorse Bioscience), by quantifying oxygen consumption rate (OCR) and extracellular acidification rate (ECAR). 7x10^3^ SW480 cells/well were seeded in XF96 culture microplates (Seahorse Bioscience, North Billerica, MA) with 100 μL complete medium, with a Viaflo Assist robot (Integra). After 24h of pre-incubation, cells were treated during 34h with OLD, GII or 6 drugs, or their controls, following the experimental design stated by Zaytseva Y. *et al.*^10^. The results were normalized against each control according to cellular confluence immediately after each assay, by scanning the plate with an IncuCyte ZOOM equipment (Essen Bioscience).

**Supplementary figures**


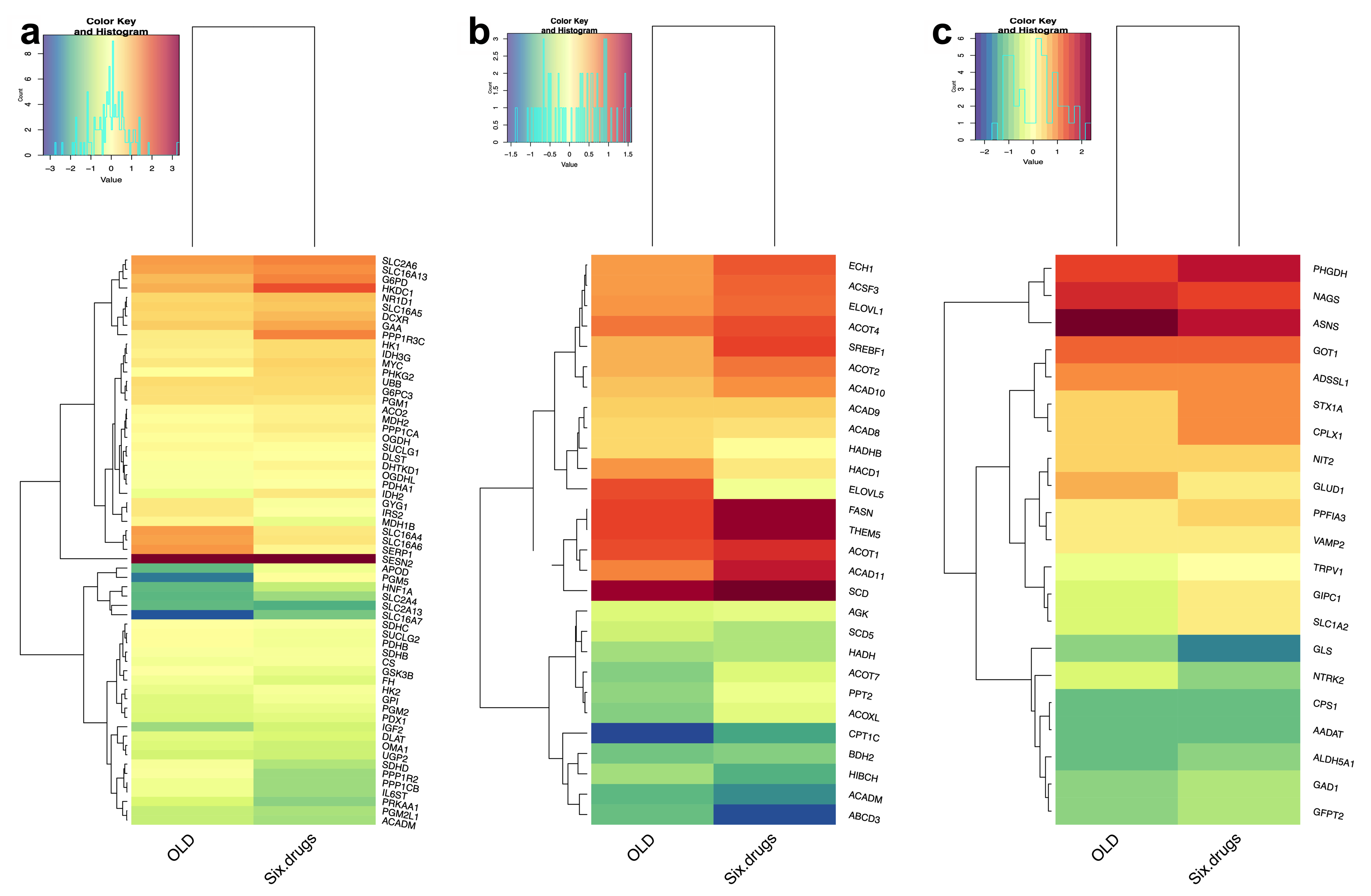


**Supplementary Figure S1.** Metabolic heatmaps after the treatment with the anti-anabolic schemes. Heatmaps of the OLD and 6 drugs conditions involving glucose and glycogen metabolism (**a**), fatty acids and β-oxidation (**b**), and glutamine metabolism (**c**). N = 3 biological replicates. *OLD: Orlistat + lonidamine + DON; 6 drugs: Orlistat + lonidamine + DON + growth hormone + insulin + indomethacin.*


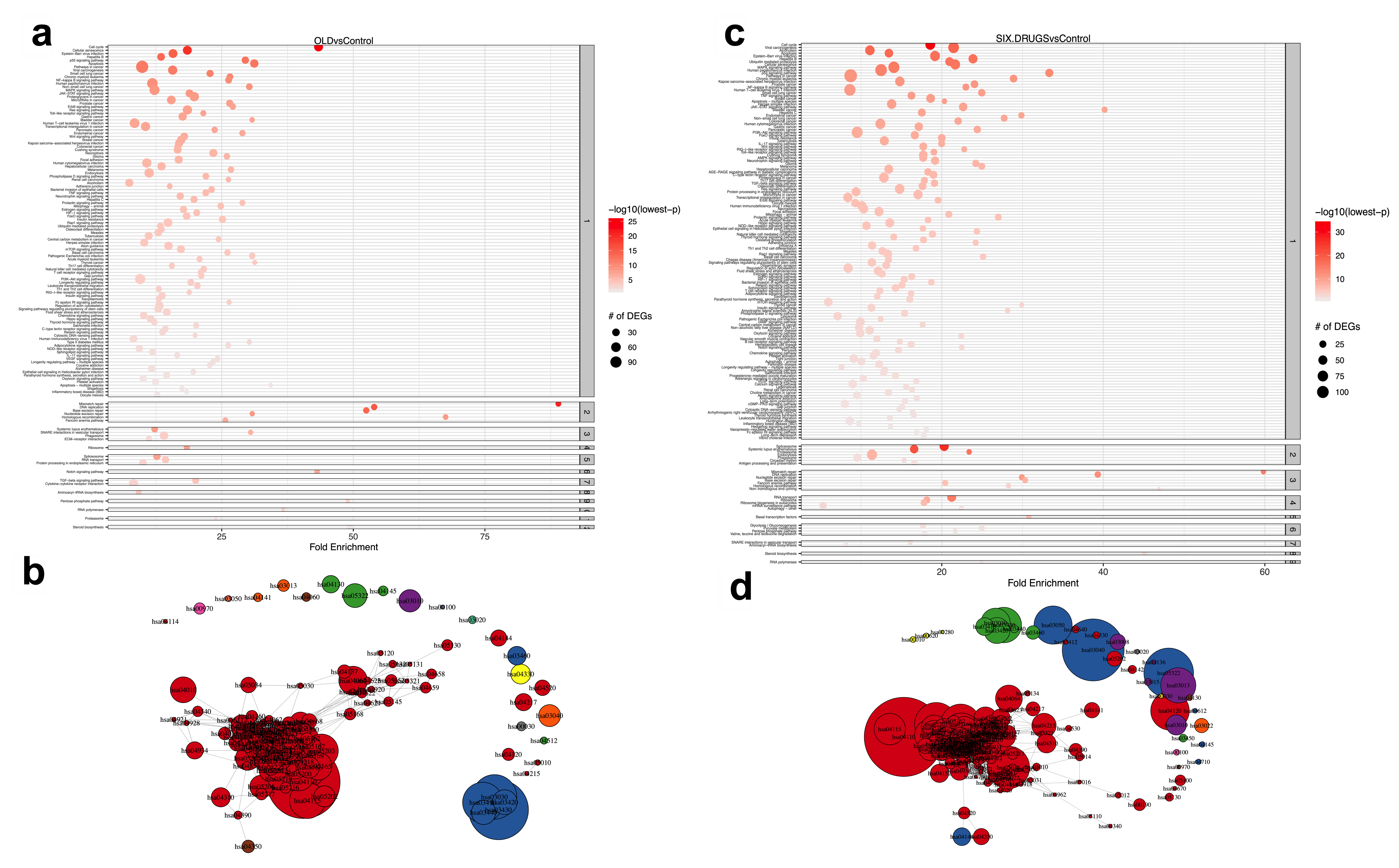


**Supplementary Figure S2.** PathfindR analysis of OLD vs control (**a-b**) and 6 drugs (**c-d**) datasets. (**a, c**) Bubble chart clustering the transcripts of the significantly altered pathways. The pathways with the lowest *p* values are shown in intense red color. The number of DEGs (# of DEGs) are schematized with the size of the orbs. The fold enrichment of genes per pathway increases from left to right. (**b, d**) Clustered pathways showing the interactions between the generated clusters in each dataset. Each color represents a cluster, being the red the most important, and the one demonstrating the biggest number of interactions. The name of each pathway is indicated with the ‘hsa’ code. For interpretation of the name of the pathways, see Supplementary Tables 4 and 5. Also, visit <https://www.genome.jp>. N = 3 biological replicates. Statistical analyses were performed with one-way analysis of variance (ANOVA) with Bonferroni correction. *OLD: Orlistat + lonidamine + DON; 6 drugs: Orlistat + lonidamine + DON + growth hormone + insulin + indomethacin; DEGs: Differentially expressed genes.*


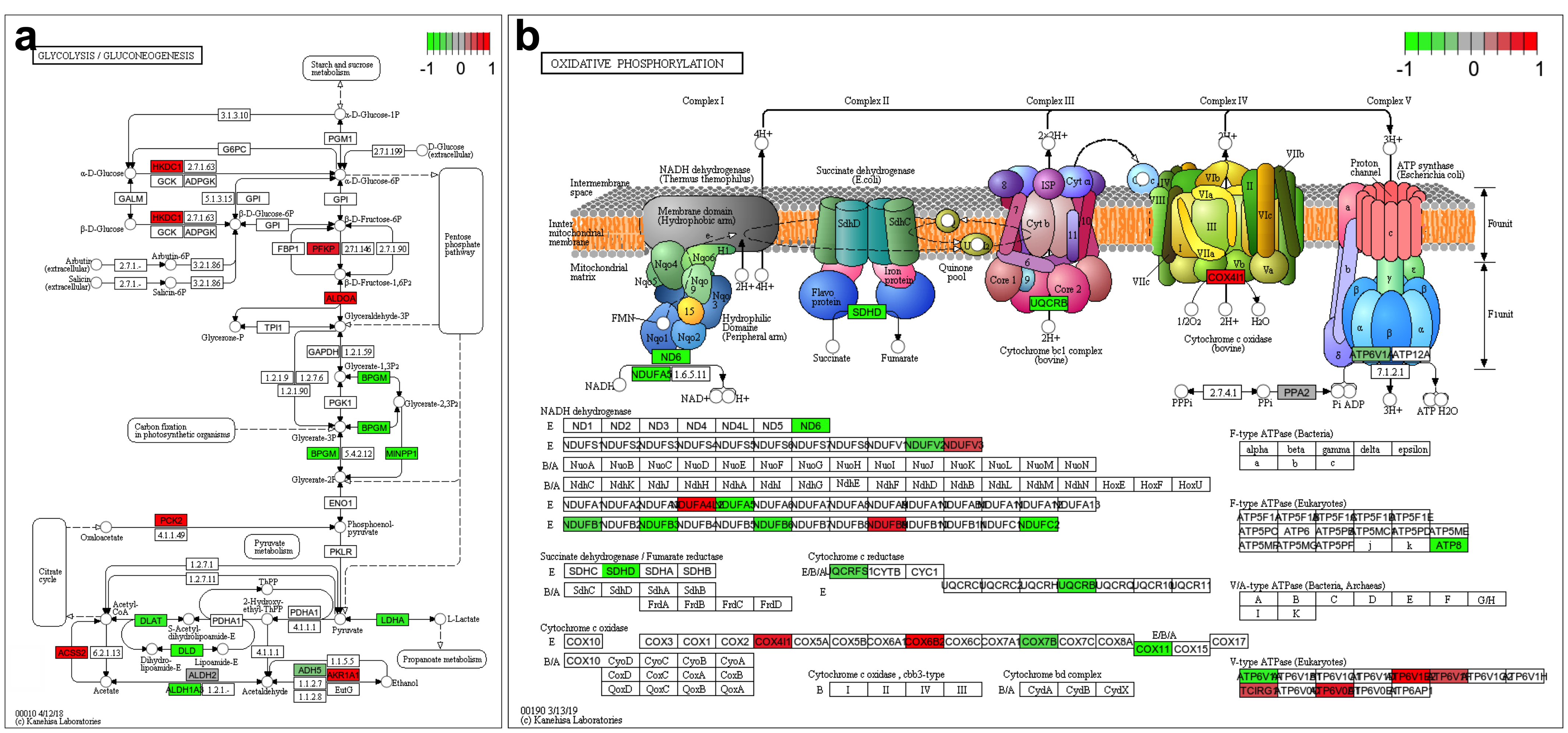


**Supplementary Figure S3.** KEGG Glycolysis/Gluconeogenesis (**a**) and oxidative phosphorylation (**b**) pathways^5-7^, showing differentially expressed genes after the treatment with 6 drugs. Upregulated genes are shown in red, and downregulated are shown in green. N = 3 biological replicates. *6 drugs: Orlistat + lonidamine + DON + growth hormone + insulin + indomethacin.*


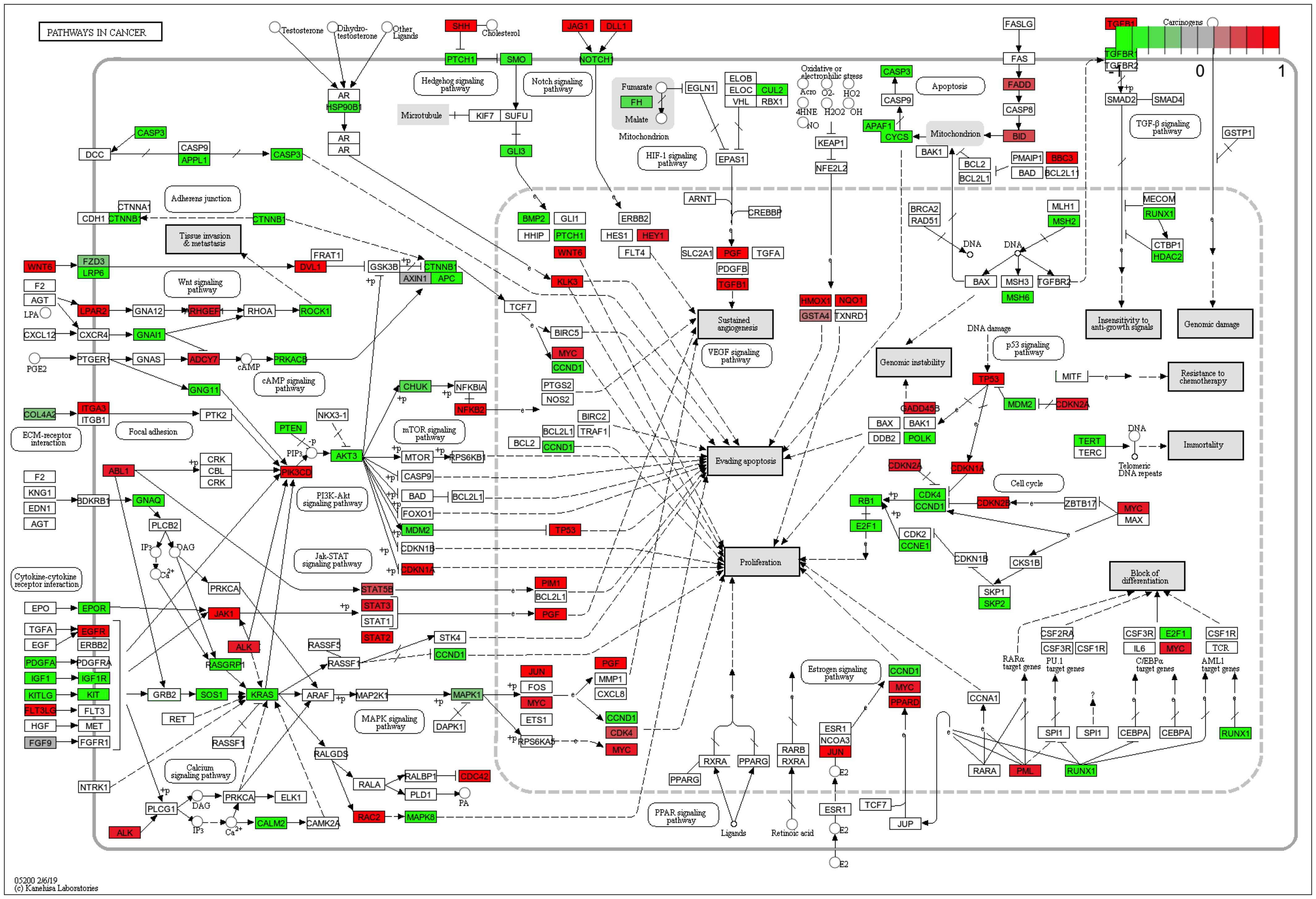


**Supplementary Figure S4.** KEGG Central carbon metabolism in cancer pathway^5-7^, showing differentially expressed genes after the treatment with 6 drugs. Upregulated genes are shown in red, and downregulated are shown in green. N = 3 biological replicates. *6 drugs: Orlistat + lonidamine + DON + growth hormone + insulin + indomethacin.*


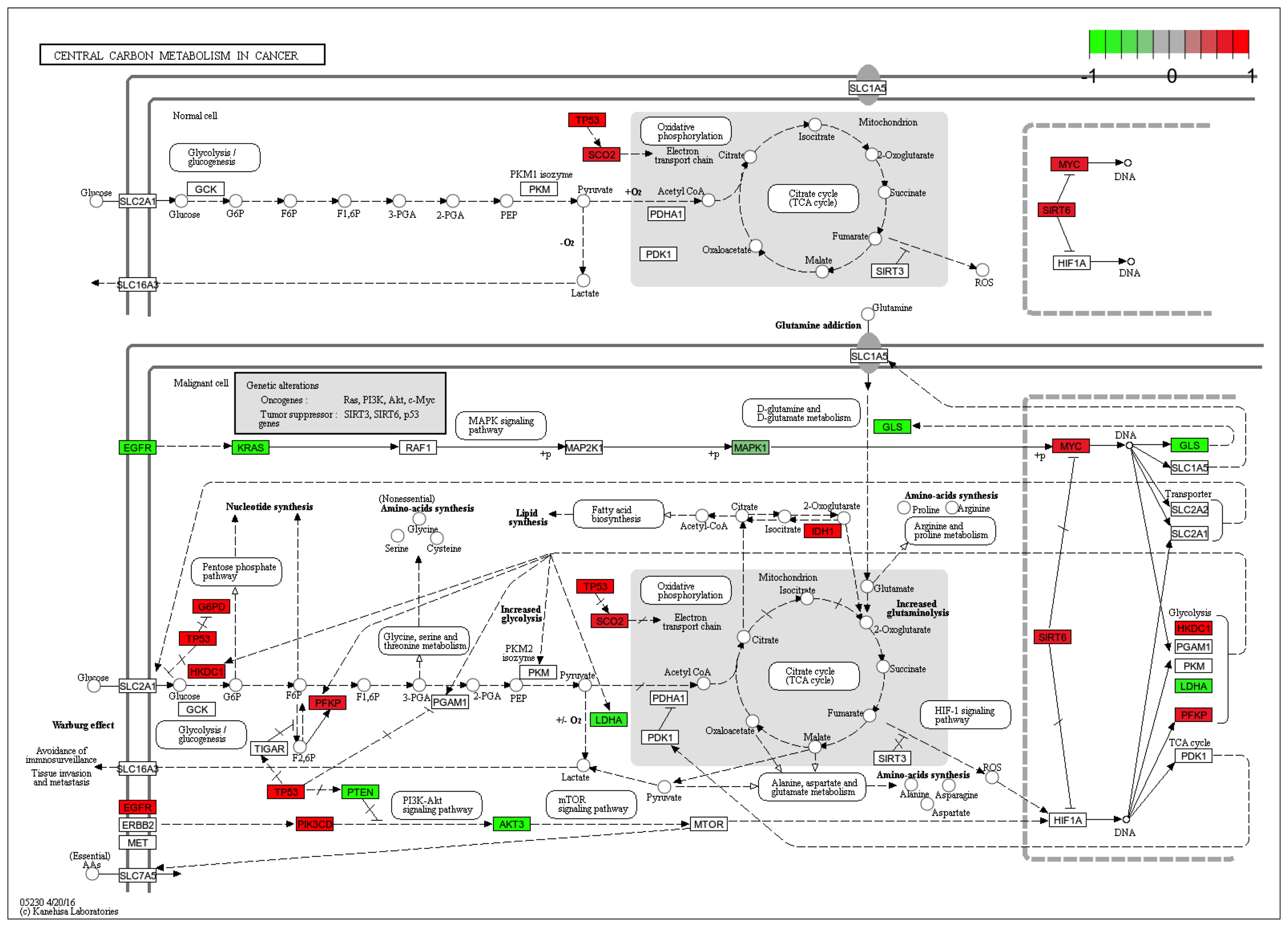


**Supplementary Figure S5.** KEGG Pathways in cancer pathway^5-7^, showing differentially expressed genes after the treatment with 6 drugs. Upregulated genes are shown in red, and downregulated are shown in green. N = 3 biological replicates. *6 drugs: Orlistat + lonidamine + DON + growth hormone + insulin + indomethacin.*


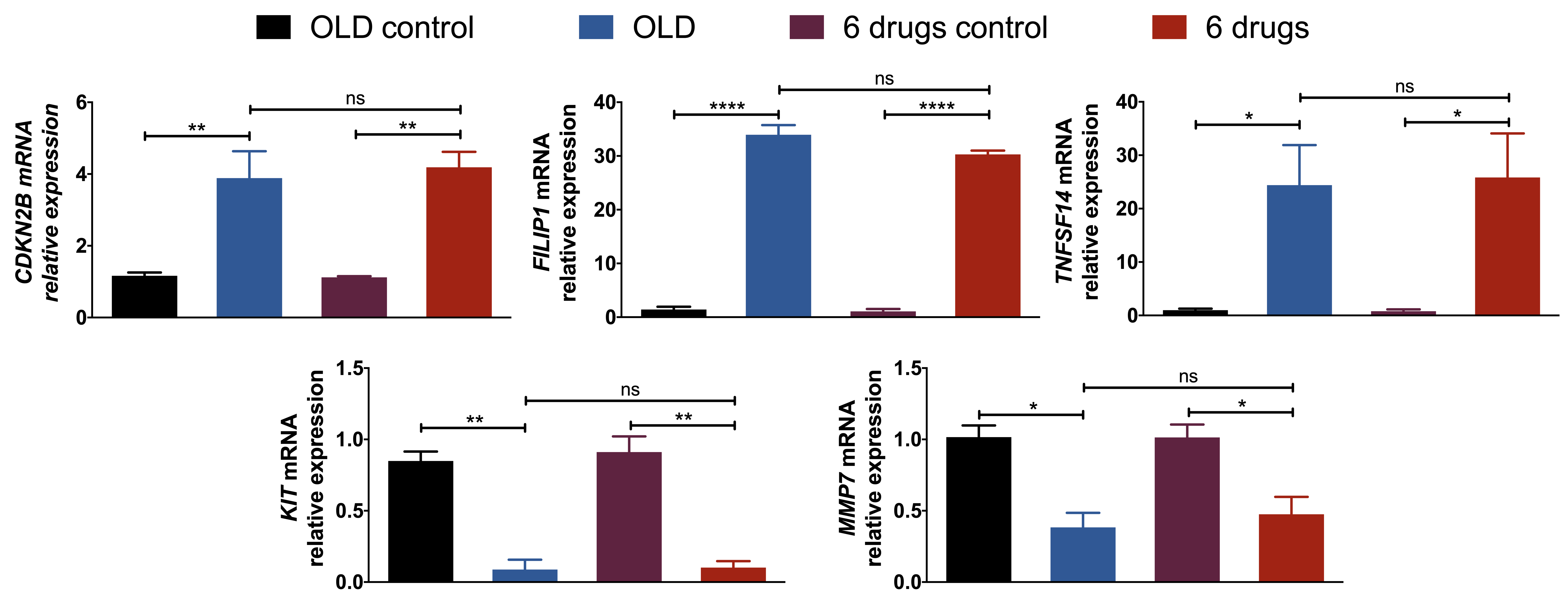


**Supplementary Figure S6.** mRNA relative expression of *CDKN2B*, *FILIP1*, *TNFSF14*, *KIT* and *MMP7*. Data are expressed as means ± s.e.m. N = 3 biological replicates. Statistical analyses were performed with one-way ANOVA with Dunnet correction. *OLD: Orlistat + lonidamine + DON; 6 drugs: Orlistat + lonidamine + DON + growth hormone + insulin + indomethacin; ns: non-significant;* **p<0.05; **p<0.01; ****p<0.0001*.


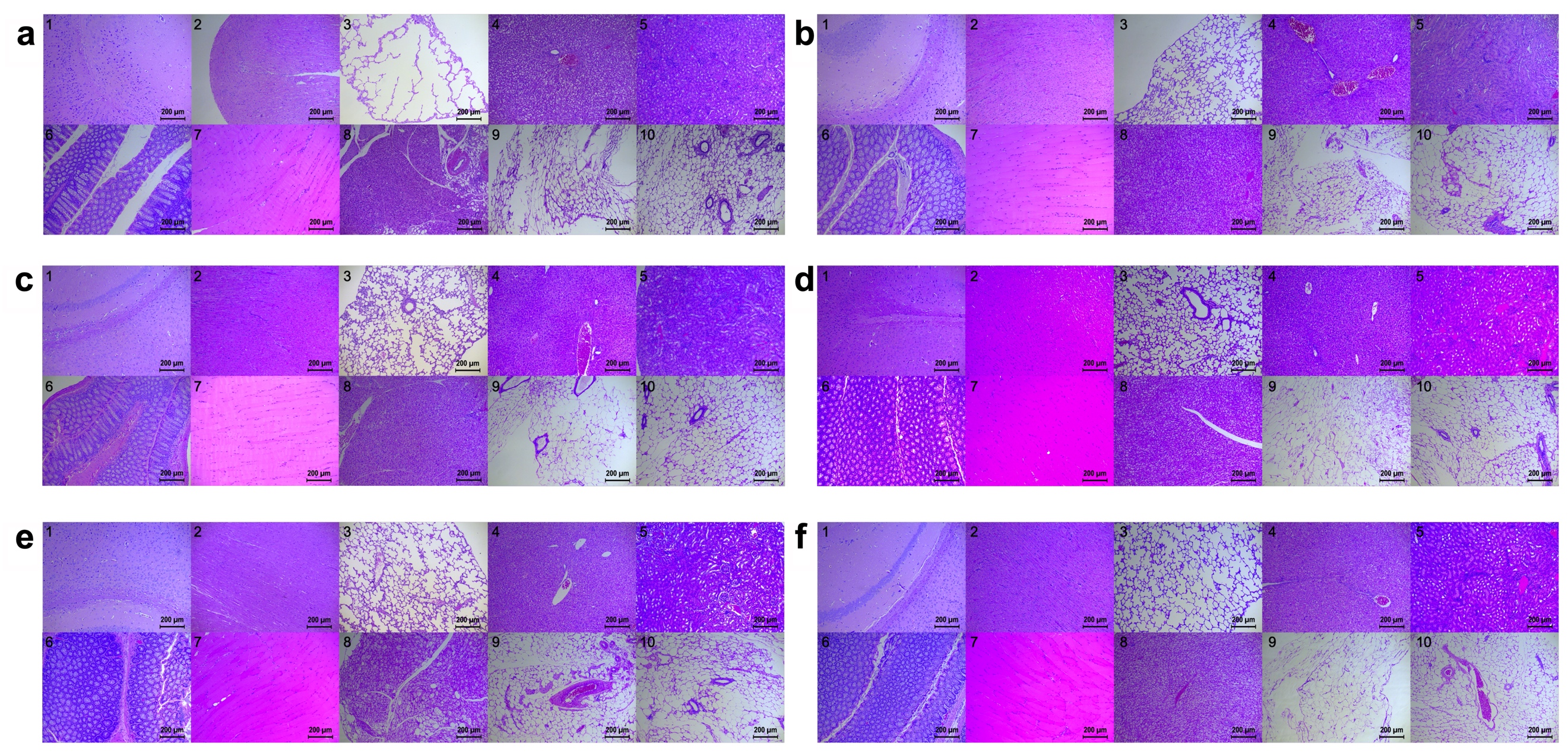


**Supplementary Figure S7.** Recovered tissues from tumor-bearing mice after 21 days of treatment. (**a**) OLD control, (**b**) OLD, (**c**) GII control, (**d**) GII, (**e**) 6 drugs control, (**f**) 6 drugs. In all the groups, there are shown brain (1), heart (2), lung (3), liver (4), kidney (5), colon (6), skeletal muscle (7), brown fat (8), visceral fat (9), and subcutaneous fat (10). H&E Images are presented at 10X magnification. The images are representative of the data obtained. Scale bars, 200 μm. N = 3 biological replicates, 8 mice/group. *OLD: Orlistat + lonidamine + DON; GII: Growth hormone + insulin + indomethacin; 6 drugs: OLD + GII*.


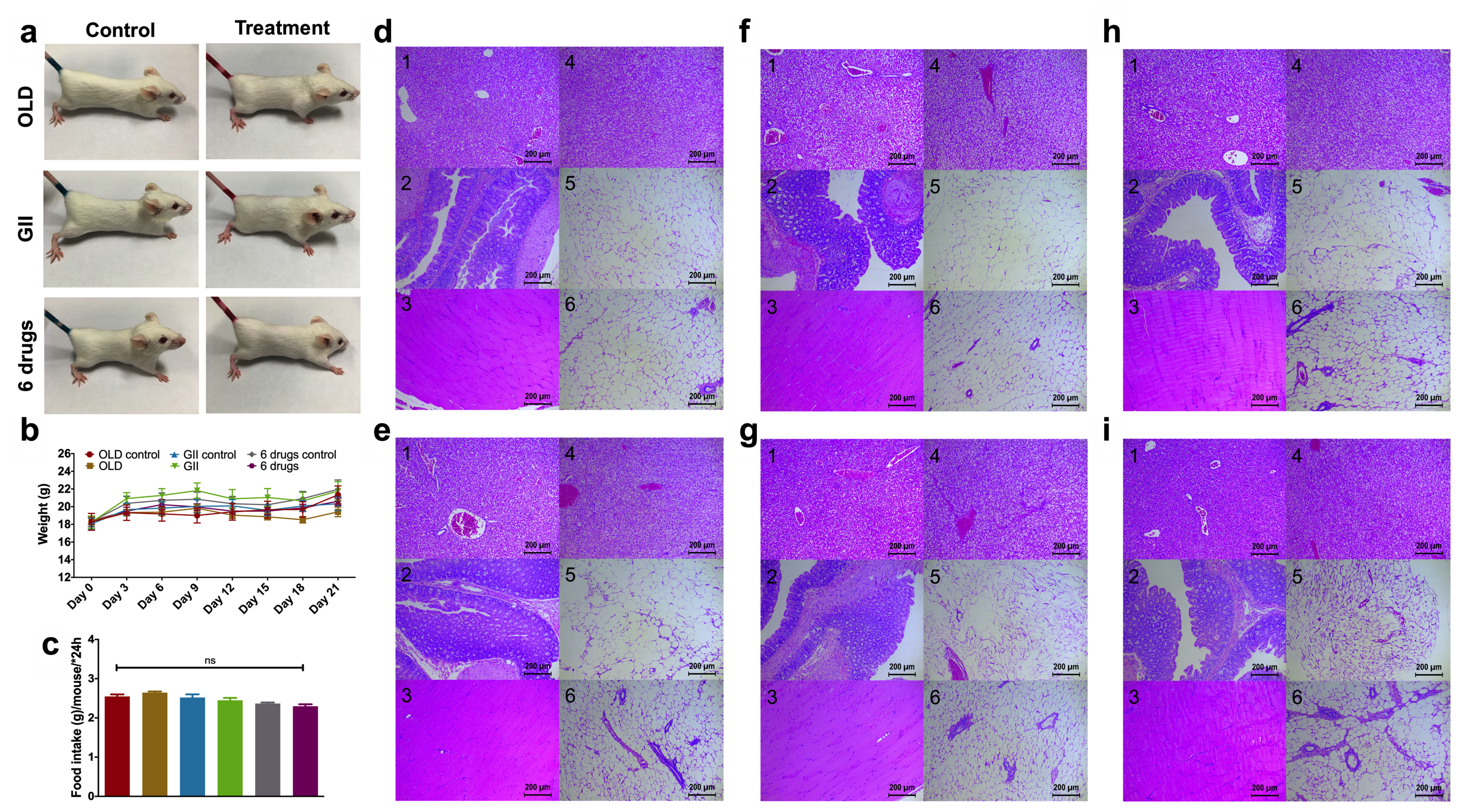


**Supplementary Figure S8.** Recovered tissues from mice without tumor after 21 days of treatment. (**a**) Photography of mice at day 21 of treatment. (**b**) Weight changes over time on the evaluated groups. (**c**) Average food intake per mouse per 24h on each group. (**d**) OLD control. (**e**) OLD. (**f**) GII control. (**g**) GII. (**h**) 6 drugs control. (**i**) 6 drugs. In all the groups, there are shown liver (1), colon (2), skeletal muscle (3), brown fat (4), visceral fat (5), and subcutaneous fat (6). H&E Images are presented at 10X magnification. The images are representative of the data obtained. N = 1 biological replicate, 10 mice/group. Statistical analysis: two-way ANOVA with Tukey correction. Data are expressed as means ± s.e.m. Scale bars, 200 μm. *OLD: Orlistat + lonidamine + DON; GII: Growth hormone + insulin + indomethacin; 6 drugs: OLD + GII; ns: non-significant*.

**Supplementary tables**

| Drug | Concentration |
| --- | --- |
| Orlistat | 8.7 µM |
| Lonidamine | 75.86 µM |
| DON | 6.12 µM |
| Growth hormone | 0.87 nM |
| Insulin | 0.809 nM |
| Indomethacin | 7.5 µM |

**Supplementary Table S1.** *In vitro* concentrations employed per compound.

| Primer target | Sequence (5' → 3') | Tm (ºC) |
| --- | --- | --- |
| *CDKN2B* | AGCGGCAGAAAGGAAAACCT | 60 |
|  | TGCCATGCGCTCAAACTAAA | 60 |
| *FILIP1* | CTATCACCATAACACCGGTCACA | 60 |
|  | GGAATGCGGGTGGGTGTAG | 60 |
| *TNFSF14* | GGGCAACCATCAGCAACAA | 60 |
|  | CCAACTGGGTCCAGGTTCCT | 60 |
| *KIT* | AAACACGGCTTAAGCAATTCCA | 60 |
|  | GCGTGTCGTTGTCTTCTTTCC | 60 |
| *MMP7* | CCCCCTGCATTTCAGGAA | 60 |
|  | TCCTGGCCCATCAAATGG | 60 |
| *HPRT1* | GAACCTCTCGGCTTTCCCG | 60 |
|  | CACTAATCACGACGCCAGGG | 60 |

**Supplementary Table S2.** Primer sequences and real-time PCR conditions for total RNA extracted from SW480 cells treated with either OLD or 6 drugs, or with their respective controls.

| Fuel test | 1º inhibitor | 2º inhibitor |  |
| --- | --- | --- | --- |
| Glucose dependency | UK5099 | BPTES+Etomoxir |  |
| Glutamine dependency | BPTES | UK5099+Etomoxir |  |
| Fatty acid dependency | Etomoxir | UK5099+BPTES |  |
| Glucose capacity | BPTES+Etomoxir | UK5099 |  |
| Glutamine capacity | UK5099+Etomoxir | BPTES |  |
| Fatty acid capacity | UK5099+BPTES | Etomoxir |  |

**Supplementary Table S3.** Inhibitors for dependency and capacity tests in the XF Mito Fuel Flex Test.

| Primer target | Sequence (5' → 3') | Tm (ºC) |
| --- | --- | --- |
| *CITED1* | TCGAGGCCTGCACTTGAT | 64 |
|  | AGCTAGTGGGAACTCCGTTG | 64 |
| *TBX1* | TGAGGAGACACGCTTCACTG | 64 |
|  | CTGCAGCGTCTTTGTCTGAG | 64 |
| *PPARγ* | CTGTGAGACCAACAGCCTGA | 64 |
|  | TCTTCCATCACGGAGAGGTC | 64 |
| *UCP1* | CTTTGCCTCACTCAGGATTGG | 64 |
|  | ACTGCCACACCTCCAGTCATT | 64 |
| *36B4* | CGACCTGGAAGTCCAACTAC | 64 |
|  | ATCTGCTGCATCTGCTTG | 64 |

**Supplementary Table S4.** Primer sequences and real-time PCR conditions for total RNA extracted from murine subcutaneous fat.

| Drug scheme | Doses | Vehicles |  | Application days |
| --- | --- | --- | --- | --- |
| OLD | Orlistat: 240 mg/kg/day^11^  Lonidamine: 0.5 mg/kg/day^11^  DON: 0.25 mg/kg/day^11^ | Orlistat: Absolute ethanol  Lonidamine: Absolute ethanol  DON: 0.9% sterile saline solution |  | 1-5, 8-12, 15-19  DON was applied only on days 1, 5 and 9^11^ |
| GII | Growth hormone: 0.5 mg/kg/day^12^  Insulin: 0.5 μg/kg/day^12^  Indomethacin: 0.5 mg/kg/day^12^ | Growth hormone: 0.9% sterile saline solution  Insulin: 0.9% sterile saline solution  Indomethacin: Absolute ethanol |  | 1-5, 8-12, 15-19 |
| 6 drugs | The same as OLD + GII | The same as OLD + GII |  | The same as OLD + GII |

**Supplementary Table S5.** Drug schemes, doses and application days of the pharmacological conditions and controls in mice. The doses and application days for the anti-anabolic scheme OLD followed the stated by Cervantes-Madrid D. and Dueñas-Gonzalez A.^11^, and the doses for the anti-catabolic scheme GII followed the stated by Chen S. and Qiu Z.^12^. *OLD: Orlistat + lonidamine + DON; GII: Growth hormone + insulin + indomethacin; 6 drugs: OLD + GII.*

| **ID** | **Pathway** | **Fold_Enrichment** | **Lowest_*p*** | **Highest_*p*** | **Up_regulated** | **Down_regulated** |
| --- | --- | --- | --- | --- | --- | --- |
| hsa04110 | Cell cycle | 43.392396 | 3.80E-26 | 1.40E-21 | ABL1, TGFB1, MYC, CDKN2B, CDKN1A, MAD1L1, TP53, GADD45A, GADD45B, SFN | CCND2, CDK6, RBL1, HDAC2, E2F1, E2F2, E2F3, TFDP1, CDKN2C, CDKN2D, CCNE1, CCNE2, CDK2, SKP2, CCNA2, CDC45, CDC7, CDK1, CCNB3, SMC1A, SMC3, STAG2, RAD21, BUB1, BUB1B, MAD2L1, CDC14B, CHEK1, CHEK2, PCNA, CDC25A, ORC1, MCM2, MCM3, MCM4, MCM5, MCM6, MCM7 |
| hsa04218 | Cellular senescence | 18.466474 | 2.80E-20 | 1.30E-11 | TGFB1, CDKN2B, PIK3CD, FOXO3, CDKN1A, HLA-B, HLA-C, HLA-E, MRAS, LIN37, MYC, TP53, GADD45A, GADD45B, SQSTM1, RELA, SERPINE1, EIF4EBP1, MAPKAPK2, ZFP36L1, CAPN2 | CDK6, CCND2, RBL1, E2F1, E2F2, E2F3, PIK3R1, CDK2, CCNE1, CCNE2, CCNA2, MYBL2, LIN9, RBBP4, RASSF5, MAP2K6, CDK1, CCNB3, NBN, CHEK2, CHEK1, CDC25A, IGFBP3, CACNA1D, CALML3, CALM3, ITPR2 |
| hsa04115 | p53 signaling pathway | 29.484287 | 3.80E-13 | 6.80E-10 | TP53, CDKN1A, SFN, GADD45A, GADD45B, CASP8, BBC3, SHISA5, AIFM2, SERPINE1, SESN2, TNFRSF10B | CHEK2, CHEK1, CCND2, CDK6, CCNE1, CCNE2, CDK2, CDK1, GTSE1, PIDD1, TP53AIP1, IGFBP3, IGF1, THBS1, TP73 |
| hsa04210 | Apoptosis | 31.184659 | 5.10E-13 | 1.30E-12 | TNFRSF10B, TNFRSF1A, TRADD, CASP8, CASP10, DIABLO, GZMB, SPTAN1, PARP3, PARP4, ERN1, CAPN2, EIF2AK3, ATF4, DDIT3, CTSB, CTSL, RIPK1, TP53, HRK, MAP3K14, RELA, GADD45A, GADD45B, BBC3, PIK3CD | TNFSF10, TUBAL3, LMNB1, LMNB2, PARP1, DFFB, ITPR2, CTSC, CTSV, PIDD1, TP53AIP1, CASP2, IL3RA, PIK3R1 |
| hsa05200 | Pathways in cancer | 9.88728 | 2.00E-12 | 2.00E-12 | AXIN1, MYC, WNT7B, ARHGEF1, ADCY7, GNG5, LAMB3, ITGA3, PIK3CD, NKX3-1, NFKB2, RELA, TP53, CDKN1A, ABL1, STAT5B, PIM1, IL6R, EPOR, ALK, JAK1, JAK3, STAT3, STAT2, STAT6, VEGFA, EGFR, FLT3LG, MET, FGF21, TFG, RAC2, PPARD, PML, CDKN2B, GADD45A, GADD45B, TGFB1, MLH1, CASP8, BBC3, HMOX1, NQO1, TXNRD1, EPAS1, JAG1, DLL1, HEY1, SHH, KLK3 | CTNNB1, CTNNA2, AXIN2, APC, WNT6, FZD3, FZD6, LRP5, LRP6, ROCK1, ROCK2, GNAI1, PRKACA, GNB3, GNG13, COL4A2, LAMA2, LAMB1, ITGA2B, PIK3R1, TRAF5, PLCB1, PLCB2, PRKCG, IL2RA, IL3RA, IL12RB2, RASGRP1, IGF1, IGF1R, KITLG, KIT, FGF3, FGF9, FGF19, SOS1, TPR, RASSF5, CALML3, CALM3, RUNX1, E2F1, E2F2, E2F3, CDK6, CCND2, SKP2, CDK2, CCNE1, CCNE2, POLK, TERT, HDAC2, MSH2, MSH6, JAG2, NOTCH1, HEYL, PTCH1, SMO, BMP2, BMP4, HSP90B1 |
| hsa04310 | Wnt signaling pathway | 17.883675 | 2.20E-07 | 3.00E-07 | WNT7B, SERPINF1, AXIN1, MYC, FOSL1, PPARD, PSEN1, TP53, TBL1X, PRICKLE3, RAC2 | WNT6, DKK4, SFRP5, FZD3, FZD6, LRP5, LRP6, BAMBI, CXXC4, CTNNB1, AXIN2, APC, CCND2, MMP7, PRKACA, GPC4, DAAM2, ROCK2, PLCB1, PLCB2, PRKCG |
| hsa05210 | Colorectal cancer | 16.663378 | 6.80E-07 | 1.10E-06 | AXIN1, MYC, PIK3CD, RAC2, TGFB1, MLH1, BBC3, TP53, CDKN1A, GADD45A, GADD45B, EREG, AREG, EGFR | AXIN2, CTNNB1, APC, PIK3R1, MSH2, MSH6, POLK, SOS1 |
| hsa04137 | Mitophagy - animal | 24.820803 | 2.00E-05 | 7.30E-05 | BCL2L13, EIF2AK3, ATF4, PINK1, UBB, SQSTM1, CALCOCO2, OPTN, NBR1, ATG9B, TFE3, BECN1, RELA, MRAS, TP53, FOXO3, RAB7A, FIS1 | TBK1, E2F1 |
| hsa05230 | Central carbon metabolism in cancer | 24.393333 | 8.10E-05 | 8.10E-05 | TP53, MYC, MET, EGFR, HKDC1, PIK3CD, IDH1, SCO2 | KIT, PGAM2, PIK3R1, SLC7A5, LDHA |
| hsa04150 | mTOR signaling pathway | 17.247811 | 9.20E-05 | 9.20E-05 | ATP6V1E2, ATP6V1F, ATP6V1H, LAMTOR1, FLCN, RRAGA, RRAGC, GRB10, EIF4EBP1, NPRL2, SEC13, SESN2, DDIT4, WNT7B, TNFRSF1A, RPS6KA2, PIK3CD, SGK1, LPIN1 | SLC7A5, RRAGB, EIF4E, SKP2, CAB39L, WNT6, FZD3, FZD6, LRP5, LRP6, IGF1, INSR, IGF1R, SOS1, PIK3R1, RICTOR, PRKCG |
| hsa04151 | PI3K-Akt signaling pathway | 9.503896 | 2.80E-04 | 2.80E-04 | EREG, AREG, FGF21, CSF1, FLT3LG, VEGFA, EGFR, MET, IL6R, EPOR, JAK1, JAK3, COL6A1, LAMB3, TNC, VWF, ITGA3, ITGA5, ITGB3, PIK3CD, GNG5, DDIT4, EIF4EBP1, PKN1, SGK1, PPP2CB, PPP2R5B, NOS3, PCK2, MYC, CDKN1A, FOXO3, ATF4, CREB3, CREB3L3, RELA, TP53 | FGF3, FGF9, FGF19, IGF1, KITLG, ERBB4, INSR, IGF1R, KIT, FLT1, SOS1, IL2RA, IL3RA, COL4A2, LAMA2, LAMB1, THBS1, ITGA2B, ITGB8, PIK3R1, GNB3, GNG13, EIF4E, SGK2, HSP90B1, PHLPP1, GYS1, CDK2, CDK6, CCND2, CCNE1, CCNE2, MYB |
| hsa04550 | Signaling pathways regulating pluripotency of stem cells | 12.935859 | 9.40E-04 | 9.40E-04 | JAK1, JAK3, STAT3, KLF4, MYC, PIK3CD, TBX3, INHBE, ACVR1B, ACVR1, WNT7B, AXIN1, JARID2, PAX6 | PIK3R1, ACVR1C, BMP4, BMPR1A, BMPR1B, ID2, WNT6, FZD3, FZD6, AXIN2, APC, CTNNB1, ESRRB, HNF1A, IGF1, IGF1R, RIF1, MEIS1, ZFHX3 |

**Supplementary Table S6.** PathfindR cancer and metabolism results of OLD vs control datasets. N = 3 biological replicates. Statistical analyses were performed with one-way analysis of variance (ANOVA) with Bonferroni correction. *OLD: Orlistat + lonidamine + DON.*

| **ID** | **Pathway** | **Fold_Enrichment** | **Lowest_*p*** | **Highest_*p*** | **Up_regulated** | **Down_regulated** |
| --- | --- | --- | --- | --- | --- | --- |
| hsa04110 | Cell cycle | 18.57233 | 2.10E-34 | 3.40E-28 | CDK4, ABL1, TGFB1, MYC, CDKN2A, CDKN2B, CDKN1A, MAD1L1, TP53, GADD45B, SFN | CCND1, CDK6, RB1, RBL1, HDAC2, E2F1, E2F3, TFDP1, CDKN2C, CCNE1, CCNE2, SKP2, CCNA2, CDC6, CDC45, CDC7, DBF4, CDK1, CCNB1, CCNB2, CCNB3, YWHAQ, YWHAE, YWHAG, WEE1, CDC27, ANAPC4, CDC23, SMC1A, SMC3, STAG2, RAD21, TTK, BUB1, BUB3, BUB1B, MAD2L1, CDC14B, CDC14A, CHEK1, PRKDC, MDM2, PCNA, CDC25A, ORC1, ORC6, MCM2, MCM3, MCM4, MCM5, MCM6, MCM7 |
| hsa04210 | Apoptosis | 13.452206 | 1.50E-18 | 5.40E-08 | TNFRSF10B, FADD, TNFRSF1A, TRADD, CASP10, BID, GZMB, SPTAN1, PARP3, ERN1, ITPR1, CAPN2, ATF4, DDIT3, CTSB, CTSL, JUN, TP53, HRK, MAP3K14, IKBKG, RELA, GADD45B, BBC3, PIK3CD | CASP3, CYCS, APAF1, LMNB1, DFFB, ITPR2, CTSC, CTSV, MAPK8, MAPK9, CHUK, PIDD1, TP53AIP1, CASP2, IL3RA, AKT3, KRAS, MAPK1 |
| hsa04218 | Cellular senescence | 21.665132 | 9.40E-17 | 1.20E-16 | TGFB1, CDKN2B, CDK4, PIK3CD, FOXO3, CDKN1A, HLA-B, HLA-C, HLA-F, HLA-E, RRAS, MRAS, LIN37, MYC, CDKN2A, TP53, GADD45B, SQSTM1, RELA, IL1A, SERPINE1, EIF4EBP1, MAPKAPK2, ZFP36L1, CAPN2, ITPR1, SLC25A6 | TGFBR1, CDK6, CCND1, RB1, RBL1, E2F1, E2F3, CCNE1, CCNE2, KRAS, AKT3, PTEN, CCNA2, LIN9, RBBP4, MDM2, HIPK3, PPP1CB, PPP1CC, MAPK1, CDK1, CCNB1, CCNB2, CCNB3, NBN, RAD1, CHEK1, CDC25A, IGFBP3, CALML3, CALM2, PPP3CB, PPP3R1, ITPR2, PPID |
| hsa04115 | p53 signaling pathway | 33.310224 | 4.10E-14 | 5.70E-14 | CDKN2A, TP53, CDKN1A, CDK4, SFN, GADD45B, BID, BBC3, SHISA5, AIFM2, SERPINE1, SESN2, STEAP3, TNFRSF10B | CHEK1, MDM2, CCND1, CDK6, CCNE1, CCNE2, CCNB1, CCNB2, CDK1, PIDD1, TP53AIP1, ZMAT3, CYCS, APAF1, CASP3, IGFBP3, IGF1, RRM2B, RRM2, SESN1, PTEN, CCNG1, TP73 |
| hsa05200 | Pathways in cancer | 8.715965 | 1.60E-12 | 2.90E-12 | AXIN1, MYC, WNT7B, FZD10, DVL3, DVL2, DVL1, LPAR2, ARHGEF1, ADCY7, LAMB3, ITGA3, PIK3CD, IKBKG, NFKB2, RELA, TP53, CDKN1A, ABL1, STAT5B, PIM1, IL6R, IL15RA, EPOR, ALK, JAK1, JAK3, STAT3, STAT2, STAT6, VEGFA, EGFR, FLT3LG, FGF21, JUN, CDK4, RAC2, PPARD, PML, CDKN2A, CDKN2B, GADD45B, TGFB1, FADD, BID, BBC3, HMOX1, NQO1, GSTM2, GSTT2B, JAG1, DLL1, HEY1, SHH, KLK3 | CASP3, APPL1, CTNNB1, AXIN2, APC, CCND1, WNT6, FZD3, LRP6, ROCK1, ROCK2, GNAI1, PRKACB, GNG11, COL4A2, LAMB1, PTEN, AKT3, CHUK, MDM2, GNAQ, IL2RA, IL3RA, IFNAR1, IFNAR2, EML4, RASGRP1, PGF, PDGFA, IGF1, IGF1R, KITLG, KIT, FGF9, FGF19, SOS1, KRAS, MAPK1, CALML3, CALM2, CDC42, MAPK8, MAPK9, RUNX1, E2F1, E2F3, CDK6, SKP2, CCNE1, CCNE2, RB1, POLK, TERT, TGFBR1, HDAC2, MSH2, MSH6, CYCS, APAF1, GSTA4, CUL2, FH, NOTCH1, HEYL, PTCH1, SMO, GLI3, BMP2, HSP90B1 |
| hsa05210 | Colorectal cancer | 22.024077 | 7.20E-10 | 8.20E-10 | AXIN1, MYC, PIK3CD, JUN, RAC2, TGFB1, BBC3, TP53, CDKN1A, GADD45B, AREG, EGFR | AXIN2, CTNNB1, APC, CCND1, KRAS, AKT3, MAPK1, MAPK8, MAPK9, CASP3, APPL1, TGFBR1, MSH2, MSH6, CYCS, POLK, SOS1 |
| hsa04151 | PI3K-Akt signaling pathway | 9.516619 | 1.80E-09 | 3.50E-09 | AREG, FGF21, CSF1, FLT3LG, VEGFA, EGFR, IL6R, EPOR, JAK1, JAK3, COL6A1, LAMB3, THBS3, TNC, VWF, ITGA3, ITGA5, ITGB3, ITGB7, PIK3CD, LPAR2, DDIT4, EIF4EBP1, PKN1, SGK1, PPP2R5B, CRTC2, NOS3, PCK2, MYC, CDKN1A, CDK4, FOXO3, ATF4, CREB3, CREB3L3, IKBKG, RELA, TP53 | FGF9, FGF19, IGF1, PDGFA, KITLG, PGF, ERBB4, IGF1R, KIT, FLT1, SOS1, KRAS, MAPK1, IL2RA, IL3RA, IFNAR1, IFNAR2, COL4A2, LAMB1, ITGB8, GNG11, PRKAA1, PRKAA2, EIF4E, PKN2, AKT3, MAGI2, PTEN, PPP2CA, PPP2R1B, PPP2R3C, PPP2R5C, PPP2R5E, HSP90B1, PHLPP1, CCND1, CDK6, CCNE1, CCNE2, YWHAQ, YWHAE, YWHAG, CREB1, CHUK, MYB, MDM2 |
| hsa04310 | Wnt signaling pathway | 17.704839 | 6.70E-09 | 1.20E-08 | WNT7B, SERPINF1, FZD10, DVL3, DVL2, DVL1, AXIN1, MYC, JUN, FOSL1, PPARD, TP53, TBL1X, PRICKLE3, RAC2 | WNT6, SFRP4, SFRP5, FZD3, LRP6, CXXC4, CTNNB1, AXIN2, APC, CCND1, MMP7, PRKACB, CACYBP, GPC4, DAAM2, ROCK2, MAPK8, MAPK9, PPP3CB, PPP3R1 |
| hsa04137 | Mitophagy - animal | 20.863863 | 1.50E-06 | 2.20E-06 | BCL2L13, ATF4, JUN, PINK1, UBB, SQSTM1, CALCOCO2, OPTN, ATG9B, TFE3, BECN1, ULK1, RELA, MRAS, RRAS, TP53, FOXO3, TBC1D17, FIS1 | MAPK8, MAPK9, RHOT1, TBK1, FUNDC1, E2F1, KRAS |
| hsa00190 | Oxidative phosphorylation | 17.738217 | 7.80E-06 | 1.20E-05 | NDUFV3, NDUFA4L2, NDUFB9, COX4I1, COX6B2, ATP6V1E2, ATP6V1F, TCIRG1, ATP6V0D1, LHPP | ND6, NDUFV2, NDUFA5, NDUFB1, NDUFB3, NDUFB6, NDUFC2, SDHD, UQCRFS1, UQCRB, COX7B, COX11, ATP8, ATP6V1A, PPA2 |
| hsa04550 | Signaling pathways regulating pluripotency of stem cells | 11.345736 | 1.60E-05 | 4.90E-04 | LIF, JAK1, JAK3, STAT3, KLF4, MYC, PIK3CD, INHBE, ACVR1, WNT7B, FZD10, DVL3, DVL2, DVL1, AXIN1, JARID2 | MAPK1, AKT3, ACVR1C, BMPR1A, BMPR1B, BMPR2, SMAD5, SMAD9, ID2, WNT6, FZD3, AXIN2, APC, CTNNB1, ESRRB, KRAS, IGF1, IGF1R, HESX1, SKIL, SMARCAD1, KAT6A, REST, RIF1, PCGF5, PCGF6 |
| hsa04150 | mTOR signaling pathway | 5.946804 | 2.00E-04 | 6.70E-04 | ATP6V1E2, ATP6V1F, LAMTOR1, FLCN, GRB10, ULK1, EIF4EBP1, NPRL2, NPRL3, WDR24, SEC13, SESN2, DDIT4, WNT7B, FZD10, DVL3, DVL2, DVL1, TNFRSF1A, RPS6KA2, PIK3CD, SGK1, LPIN1 | ATP6V1A, FNIP2, EIF4E, SKP2, SEH1L, CAB39, CAB39L, PRKAA1, PRKAA2, WNT6, FZD3, LRP6, IGF1, IGF1R, SOS1, KRAS, MAPK1, RPS6KA3, PTEN, AKT3, CHUK, RICTOR |
| hsa05230 | Central carbon metabolism in cancer | 17.633735 | 4.50E-04 | 5.60E-04 | TP53, SIRT6, MYC, EGFR, G6PD, HKDC1, PFKP, PIK3CD, IDH1, SCO2 | KIT, KRAS, MAPK1, GLS, PTEN, AKT3, LDHA |
| hsa00010 | Glycolysis / Gluconeogenesis | 17.676329 | 2.90E-03 | 5.30E-03 | HKDC1, PFKP, ALDOC, ALDOA, AKR1A1, ALDH2, ALDH3B1, ACSS1, ACSS2, PCK2 | DLAT, DLD, LDHA, ADH5, ALDH3A2, ALDH1A3, BPGM, MINPP1 |
| hsa00620 | Pyruvate metabolism | 24.954817 | 6.50E-03 | 1.10E-02 | ACSS1, ACSS2, ALDH2, PC, PCK2, ACAT2 | DLAT, DLD, ACYP1, ALDH3A2, LDHA, GLO1, ME2, MDH1, FH, ACAT1 |

**Supplementary Table S7.** PathfindR cancer and metabolism results of 6 drugs vs control datasets. N = 3 biological replicates. Statistical analyses were performed with one-way analysis of variance (ANOVA) with Bonferroni correction. *6 drugs: Orlistat + lonidamine + DON + growth hormone + insulin + indomethacin.*

**References**

1 Bolger, A. M., Lohse, M. & Usadel, B. Trimmomatic: a flexible trimmer for Illumina sequence data. *Bioinformatics* **30**, 2114-2120, doi:10.1093/bioinformatics/btu170 (2014).

2 Liao, Y., Smyth, G. K. & Shi, W. The R package Rsubread is easier, faster, cheaper and better for alignment and quantification of RNA sequencing reads. *Nucleic Acids Res* **47**, e47, doi:10.1093/nar/gkz114 (2019).

3 Robinson, M. D., McCarthy, D. J. & Smyth, G. K. edgeR: a Bioconductor package for differential expression analysis of digital gene expression data. *Bioinformatics* **26**, 139-140, doi:10.1093/bioinformatics/btp616 (2010).

4 Ritchie, M. E. *et al.* limma powers differential expression analyses for RNA-sequencing and microarray studies. *Nucleic Acids Res* **43**, e47, doi:10.1093/nar/gkv007 (2015).

5 Kanehisa, M. G., S. KEGG: kyoto encyclopedia of genes and genomes. *Nucleic Acids Res* **28**, 27-30, doi:10.1093/nar/28.1.27 (2000).

6 Kanehisa, M. Toward understanding the origin and evolution of cellular organisms. *Protein Sci* **28**, 1947-1951, doi:10.1002/pro.3715 (2019).

7 Kanehisa, M. F., M.; Sato, Y.; Ishiguro-Watanabe, M.; Tanabe, M. KEGG: integrating viruses and cellular organisms. *Nucleic Acids Res* **49**, D545-D551, doi:10.1093/nar/gkaa970 (2021).

8 Ulgen, E. O., O.; Sezerman, O. U. pathfindR: An R Package for Pathway Enrichment Analysis Utilizing Active Subnetworks. *bioRxiv*, 272450, doi:doi.org/10.1101/272450 (2018).

9 Luo, W. & Brouwer, C. Pathview: an R/Bioconductor package for pathway-based data integration and visualization. *Bioinformatics* **29**, 1830-1831, doi:10.1093/bioinformatics/btt285 (2013).

10 Zaytseva, Y. Y. *et al.* Increased expression of fatty acid synthase provides a survival advantage to colorectal cancer cells via upregulation of cellular respiration. *Oncotarget* **6**, 18891-18904, doi:10.18632/oncotarget.3783 (2015).

11 Cervantes-Madrid, D. & Duenas-Gonzalez, A. Antitumor effects of a drug combination targeting glycolysis, glutaminolysis and de novo synthesis of fatty acids. *Oncol Rep* **34**, 1533-1542, doi:10.3892/or.2015.4077 (2015).

12 Chen, S. Z. & Qiu, Z. G. Combined treatment with GH, insulin, and indomethacin alleviates cancer cachexia in a mouse model. *J Endocrinol* **208**, 131-136, doi:10.1677/JOE-10-0341 (2011).
